# Bruceae Fructus Oil Inhibits Triple-Negative Breast Cancer by Restraining Autophagy: Dependence on the Gut Microbiota-Mediated Amino Acid Regulation

**DOI:** 10.3389/fphar.2021.727082

**Published:** 2021-10-01

**Authors:** Jiyan Su, Xiaohong Chen, Yuanjie Xiao, Dan Li, Muxia Li, Hongfu Li, Jiangjian Huang, Zhengquan Lai, Ziren Su, Yizhen Xie, Dajiang Zhu, Qianjun Chen, Hai Lu, Jingjin He, Chenglai Xia

**Affiliations:** ^1^ Affiliated Foshan Maternity and Child Healthcare Hospital, Southern Medical University, Foshan, China; ^2^ Department of Basic Medical Science, Xiamen Medical College, Xiamen, China; ^3^ Guangdong Provincial Key Laboratory of Microbial Safety and Health, State Key Laboratory of Applied Microbiology Southern China, Guangdong Institute of Microbiology, Guangdong Academy of Science, Guangzhou, China; ^4^ Department of Cell Biology and Institute of Biomedicine, College of Life Science and Technology, Jinan University, Guangzhou, China; ^5^ School of Pharmaceutical Science, Guangzhou University of Chinese Medicine, Guangzhou, China; ^6^ The Eighth Affiliated Hospital, Sun Yat-sen University, Shenzhen, China; ^7^ Guangzhou Baiyunshan Ming Xing Pharmaceutical Co., Ltd., Guangzhou, China; ^8^ Department of Pharmacy, Shenzhen University General Hospital, Shenzhen University, Shenzhen, China; ^9^ Department of Breast Disease, Guangdong Provincial Hospital of Chinese Medicine, Guangzhou, China; ^10^ Shenzhen International Institute for Biomedical Research, Shenzhen, China; ^11^ School of Pharmaceutical Sciences, Southern Medical University, Guangzhou, China

**Keywords:** Bruceae fructus oil, triple-negative breast cancer, gut microbiota, amino acid metabolism, autophagy, mTOR

## Abstract

Triple-negative breast cancer (TNBC) has been acknowledged as an aggressive disease with worst prognosis, which requires endeavor to develop novel therapeutic agents. Bruceae fructus oil (BO), a vegetable oil derived from the fruit of *Brucea javanica* (L.) Merr., is an approved marketable drug for the treatment of cancer in China for several decades. Despite that the anti–breast cancer activity of several quassinoids derived from *B. javanica* has been found, it was the first time that the potential of BO against TNBC was revealed. Although BO had no cytotoxicity on TNBC cell lines *in vitro*, the oral administration of BO exhibited a gut microbiota–dependent tumor suppression without toxicity on the non-targeted organs *in vivo*. By metagenomics and untargeted metabolomics, it was found that BO not only altered the composition and amino acid metabolism function of gut microbiota but also regulated the host’s amino acid profile, which was in accordance with the metabolism alternation in gut microbiota. Moreover, the activity of mTOR in tumor was promoted by BO treatment as indicated by the phosphorylation of 4E-binding protein 1 (4E-BP1) and ribosomal protein S6, and hyper-autophagy was consequently restrained. By contrast, the failure of tumor suppression by BO under pseudo germ-free (PGF) condition came with indistinctive changes in autophagy and mTOR activity, implying the critical role of the gut microbiota in BO’s anticancer activity. The present study highlighted a promising application of BO against breast cancer with novel efficacy and safety.

## Introduction

As the leading cause of cancer-related deaths in female individuals, breast cancer has been more and more alarming due to its rise in incidence among younger adults (Bray et al., 2018). Nowadays, chemotherapy, endocrinotherapy, and targeted therapy are the preoccupant drug regimens against breast cancer in addition to surgery and radiotherapy. Nevertheless, compared with other subtypes of breast cancer, triple-negative breast cancer (TNBC) has been acknowledged as an aggressive disease with worst prognosis due to the unavailability of endocrinotherapy or targeted therapy options. Despite that immunotherapies have been tested in some clinical trials with encouraging results (Franzoi et al., 2020), the recommended systemic treatment for TNBC is mainly focused on chemotherapy, including alkylating agents, antibiotics, anthracyclines, and taxanes (Waks and Winer, 2019; Lee J. S. et al., 2020). Unfortunately, in addition to resistance, these common remedies are always accompanied by various side effects that cause systemic multi-organ toxicity, such as myelosuppression, cardiotoxicity, hepatotoxicity, and renal toxicity. Therefore, endeavors are still required to develop novel therapeutic agents to improve the prognosis of TNBC.

Gut microbiota, together with its versatile effects on the host, has exciting implications for cancer prognosis and therapy. Accumulating evidence has revealed that dysbiosis of gut microbiota is closely related to cancer progression, such as colon cancer (Lau et al., 2021), liver cancer (Schwabe and Greten, 2020), pancreatic cancer (Pan et al., 2020), and breast cancer (Zhu et al., 2018). On the other hand, it is a breakthrough discovery that gut microbiota plays a crucial role in response to immunotherapy (Baruch et al., 2020), chemotherapy (Daillère et al., 2016), radiotherapy (Guo et al., 2020), and targeted therapy (Di Modica et al., 2021). As research moves along, it has been found that the impact of gut microbiota on cancer progression and therapy response would be attributed to some specific bacteria, such as *Akkermansia muciniphila* (Routy et al., 2018) and *Fusobacterium nucleatum* (Slade, 2020), and their metabolites would serve as the functional medium, including short-chain fatty acids and bile acid. Hence, gut microbiota has been proposed as the promising target for therapeutic regimen and diagnosis, but the underlying mechanism remains to be explored.

*Bruceae fructus* (named Ya-dan-zi in Chinese) is the fruit of *Brucea javanica* (L.) Merr., which is widely distributed throughout Southeastern Asia and northern Oceania (Dong et al., 2013). In traditional Chinese medicine, *B. javanica* has been used in the treatment against dysentery (2020), malaria (Kefe et al., 2016), and inflammation (Yang et al., 2013) for thousands of years. Anticancer activity has now been a hot spot for the study and clinical application of *B. javanica*. It has been found that quassinoids derived from *B. javanica*, such as brucein D and brusatol, displayed potent anticancer activities against pancreatic cancer (Lai et al., 2017), osteosarcoma (Wang S. et al., 2019), and breast cancer (Mohan et al., 2021). And the underlying mechanisms possibly involve reactive oxygen species (ROS) regulation (Xie et al., 2019), phosphoinositide 3-kinase (PI3K)/Akt signaling pathway (Lai et al., 2017), JAK-STAT signaling pathway (Wang S. et al., 2019), and so on. Bruceae fructus oil (BO) is a vegetable oil derived from the fruit of *Brucea javanica* (L.) Merr. However, only BO has been approved as a marketable drug so far, which is mainly in the form of oral emulsion, parenteral emulsion, and soft capsules, for the auxiliary treatment of liver cancer (Luo et al., 2020), gastrointestinal cancer (Yan et al., 2015; Wu et al., 2018), lung cancer, and brain metastasis of lung cancer (Zhang et al., 2018). It is well known that the composition of BO is similar to most of the other cooking oils (such as olive oil, coix seed oil, sunflower seed oil, and peanut oil) (Tian et al., 2016), almost composed of fatty acids, such as oleate, linoleate, and palmitate. Given that these fatty acids would hardly have direct cytotoxicity on cancer cells, it is still a hot affair to explore the active constituents responsible for the anticancer activity of BO and how they work. Recently, we found that BO exhibited a stunning inhibition on TNBC in a mice xenograft tumor model, and interestingly, this suppression depends on the existence of gut microbiota. Hence, the present study aims to investigate the underlying mechanism of BO against TNBC and the indispensability of gut microbiota in it.

## Materials and Methods

### Reagents and Chemicals

Bruceae fructus oil emulsion (BO) was provided by Guangzhou Baiyunshan Mingxing Pharmaceutical Co., Ltd. (Guangzhou, China). The composition of BO was quantified by gas chromatography–mass spectrometry (GC-MS), and the details are given in the supplementary material. Paclitaxel injection (PTX) was obtained from Hainan Quanxing Pharmaceutical Co., Ltd. (Hainan, China). Olive oil, oleate, linoleate, and palmitate were from Shanghai Macklin Biochemical Co., Ltd. (Shanghai, China). Ampicillin sodium, metronidazole, neomycin sulfate, and vancomycin hydrochloride were purchased from Shanghai Aladdin Biochemical Technology Co., Ltd. (Shanghai, China). Details about primary and secondary antibodies are listed in supporting information Supplementary Table S4.

### Animals

Female Balb/c nude mice (3–4 weeks old) were purchased from the Guangdong Medical Laboratory Animal Center (Foshan, China) and the Laboratory Animal Center, Southern Medical University (Guangzhou, China). All animals were acclimatized under the condition of controlled temperature (23 ± 2°C), humidity (50 ± 5%), and 12-h light/dark cycle, and they were given free access to food and water. All experiments were performed after the 7-day acclimatization, and they were approved by the Guangdong Institute of Microbiology Laboratory Animal Ethics Committee according to the guidelines (permission numbers: GT-IACUC201807262 and GT-IACUC202005111), in accordance with the Guidelines for the Care and Use of Laboratory Animals published by the U.S. National Institutes of Health (NIH Publication, revised 2011).

### Cell Culture

Human TNBC cell lines MDA-MB-231 and BT-20 were provided by the Cell bank of Chinese Academy of Sciences (Shanghai, China) and American Type Culture Collection (United States). MDA-MB-231 and BT-20 cells were cultured in DMEM (4.5 mg/ml D-glucose, Gibco, NY, United States) supplemented with 10% fetal bovine serum (FBS, Gibco, NY, United States) and 1% penicillin/streptomycin (Gibco, NY, United States), and maintained in an incubator at 37°C under an atmosphere of 5% CO_2_.

### Cytotoxicity Assay

The cells were inoculated in 96-well plates at a density of 3 × 10^4^ cells/mL and treated with the corresponding drugs. After 48 h, 20 μL of Cell Titer 96®AQueous MTS Reagent (Promega, Wisconsin, United States) was added to each well, and the optical density was measured at 490 nm on a Multiscan MK3 microplate reader (Thermo Fisher, United States) after 4 h.

### TNBC Model Induction and Treatment

For the experiment under specific pathogen-free (SPF) condition, MDA-MB-231 cells were subcutaneously (*s.c.*) injected into the right foreleg armpit of the BALB/c nude mice (0.1 ml/mouse, 2 × 10^6^ cells/mouse). After the tumor grew to ∼1 mm^3^, the mice were randomly divided into five groups (6 mice in each group), including the model group, PTX group, and BO groups. In the following 30 days, the mice in BO groups were orally administered with BO (100, 200, and 400 mg/kg) once a day, while those in the PTX group were intraperitoneally (*i. p.*) injected with PTX (12.5 mg/kg) twice a week. The model group mice were orally given equal volumes of distilled water.

For the experiment under pseudo germ-free (PGF) condition, the mice that grew up under SPF condition were housed in gnotobiotic facility, and the bedding, feed, and all the experiment apparatus were sterilized by Co^60^ radiation. Prior to the TNBC model induction and treatment, the mice were orally administered with broad-spectrum antibiotics for 7 days, once a day. The broad-spectrum antibiotics included ampicillin sodium (200 mg/kg), metronidazole (200 mg/kg), neomycin sulfate (200 mg/kg), and vancomycin hydrochloride (200 mg/kg). Then they were subjected to model induction. After the tumor had grown to ∼1 mm^3^, the mice were randomly divided into the ABX model group and ABX-BO group, with eight mice in each. The mice in the ABX-BO group and ABX model group were orally administered with 400 mg/kg BO and equal volumes of distilled water, respectively, for 20 days.

The body weight and tumor volume were monitored twice a week. The volume was calculated as V = *a*×*b* ^2^/2, where *a* indicates the longer diameter and *b* indicates the shorter diameter. On the next day of the last administration, peripheral blood was collected from the orbital vein plexus. The tumor, small intestine, liver, and cecal contents were harvested after the mice were killed by cervical dislocation. Tumors were weighed, photographed, and segmented.

### Hematoxylin–Eosin Staining

Parts of the small intestine and liver were fixed in 4% paraformaldehyde, embedded in paraffin, sliced into 3-μm-thick sections, and stained with hematoxylin–eosin (H&E). The slides were observed under a light microscope with 200 × magnification.

### Western Blot Analysis

Proteins from the tumor tissue were extracted with a tissue total protein extraction kit (SolarBio Tech Co., Ltd., Beijing, China) by homogenization at 4°C, and they were quantified by a bicinchoninic acid (BCA) protein assay kit (CoWin Biosciences Co., Ltd., Beijing, China); 30 μg proteins were separated by polyacrylamide gel electrophoresis and then transferred onto a PVDF membrane. Subsequently, the membrane was blocked with 5% skim milk in TBST for 1 h and incubated with the primary antibodies overnight at 4°C and with secondary antibodies for 1 h the next day. The protein bands were detected with the enhanced chemiluminescence (ECL) detection reagents (SolarBio Tech Co., Ltd., Beijing, China), and the band intensity was quantified using ImageJ software (NIH Image, United States).

### Real-Time Quantitative Polymerase Chain Reaction (RT-qPCR) Analysis

Total RNAs from tumor tissues were extracted with TRIzol (Thermo Fisher Scientific, NY, United States) by homogenization at 4°C according to the manufacturer’s instruction; 3 μg of total RNA was reversed to cDNA with a ReverAid First Strand cDNA Synthesis Kit (Thermo Scientific, MA, United States). RT-qPCR reactions were performed with SYBR^®^ Premix Ex Taq™ II (Takara Bio, Shiga, Japan) using the Step One Plus Real-Time PCR system (Thermo Fisher Scientific, NY, United States). The primer sequences are shown in Supporting Information Supplementary Table S5.

### Metagenome Analysis for Microbiota in Cecal Contents

Metagenome analysis was performed by Personal Biotechnology Co., Ltd. (Shanghai, China). Total microbial genomic DNAs from cecal content were extracted with the DNeasy PowerSoil Kit (QIAGEN, Inc., Netherlands) according to the manufacturer’s instructions. The extracted DNAs were quantified and subjected to integrity check by agarose gel electrophoresis. The qualified DNA samples were used to construct metagenome shotgun sequencing libraries with insert sizes of 400 bp with the Illumina TruSeq Nano DNA LT Library Preparation Kit (Illumina, United States). Each library was sequenced on the Illumina HiSeq X-ten platform (Illumina, United States) with PE150 strategy.

Raw sequencing reads were subjected to quality filtration prior to the further analysis. Sequencing adapters were removed by Cutadapt (v1.2.1), and the low-quality reads were trimmed by a sliding window algorithm. The reads were aligned to the host genome by BWA (http://bio-bwa.sourceforge.net/) (Li and Durbin, 2009) to eliminate host contamination. The quality-filtered reads were assembled *de novo* to construct the metagenome for each sample by megahit (https://hku-bal.github.io/megabox/). All coding regions (CDSs) of metagenomic scaffolds longer than 300 bp were predicted by MetaGeneMark (http://exon.gatech.edu/GeneMark/metagenome). The CDS sequences of all samples were clustered by CD-HIT at 90% protein sequence identity, to obtain a non-redundant gene catalog. Gene abundance in each sample was estimated by soap.coverage (http://soap.genomics.org.cn/) based on the number of aligned reads. The lowest common ancestor taxonomy of the non-redundant genes was obtained by aligning them against the NCBI-NT database by BLASTN (e value < 0.001). Similarly, the functional profiles of the non-redundant genes were obtained by annotation against the GO and KEGG databases, respectively, by using DIAMOND alignment algorithm.

Based on the data of taxonomic and functional profile analysis, *α*-diversity analysis was evaluated by ACE, Chao1, Simpson, and Shannon indexes. *β*-diversity analysis was performed to compare the composition and function of microbial communities across samples using Bray–Curtis distance metrics and visualized *via* principal coordinate analysis (PCoA). *β*-diversity variation was further confirmed by the analysis of similarities (ANOSIM). LEfSe (linear discriminant analysis effect size) was employed to figure out differentially abundant taxa and functions across groups using the default parameters. Function comparison and enrichment according to KEGG were performed on MicrobiomeAnalyst (https://www.microbiomeanalyst.ca) (Dhariwal et al., 2017; Chong et al., 2020)

### Untargeted Metabolomics for Serum

The serum was collected and stored at −80°C for metabolomics analysis. After thawed on ice, the samples were obtained through ultrasonic extraction with 400 μL methothol/acetonitrile (1:1, v/v) for 10 min. After protein precipitation for 1 h at −20°C, the samples were centrifuged (15 min, 4°C, 12,000 *rcf*), and the supernatant was transferred to a new tube and dried with Termovap sample concentrator. The extract was reconstituted in acetonitrile/water (1:1, v/v) by ultrasound for 5min and centrifuged (15 min, 4°C, 12000 *rcf*), and the supernatant was collected and analyzed with Q Exactive Orbitrap ultrahigh performance liquid chromatography–tandem mass spectroscopy (UPLC-MS/MS, Thermo Fisher Scientific, Waltham, MA, United States). The mass spectroscopy was equipped with electrospray ionization (ESI) in positive and negative ion modes, which was controlled by Thermo Xcalibur 3.0.63 (Thermo Fisher Scientific, Waltham, MA, United States).

Chromatography analysis was performed on an ACQUITY UPLC HSS T3 column (Waters, Milford, United States, 100 Å, 1.8 µm, 2.1 mm × 100 mm) at 30°C. The flow rate was 0.3 ml/min. The mobile phase was composed of 0.1% formic acid (A) and acetonitrile (B), and the gradient elution program was as follows: 0.01–1.5 min, 99–99% A; 1.5–13 min, 99→1% A; 13–16.5 min, 1→1% A; 16.5–16.6 min, 1→99% A; 16.6–20 min, 99→ 99% A. The mass spectrometry analysis condition was as follows: the ion source detection modes were ESI+ and ESI, respectively; the ion source voltage was +4.5 kV and −4.5 kV; the scanning range was m/z 50–1,500; the resolving power for full-scan MS and MS^2^ data collection was 70,000 and 17,500 M, respectively; and the collision energies (CE) were 20, 40, 60 eV, respectively.

The original mass spectrometry data obtained by UPLC-MS/MS were processed by Compound Discover 3.0 software (Thermo Fisher Scientific, Waltham, MA, United States). The metabolites were annotated with the obtained retention time, peak area, and ion fragment characterization (m/z value and abundance), followed by identification with reference. The data result sets containing all the m/z value, retention time, and ion peak area of each sample were exported to SIMCA (version 14.0, Umetrics, Umea, Sweden) for differential metabolites selection according to the VIP value (>1) and *p* value (<0.05).

### Statistical Analysis

All data were expressed as mean ± standard deviation (SD). Statistical analysis was performed with Statistical Package for the Social Sciences (SPSS 22.0, Chicago, United States). First, datasets from each experiment were subjected to the normal distribution test . If it complied with normal distribution, the data were analyzed by one-way analysis of variance (ANOVA) or Student’s *t* test, and the relationship between microbes and metabolites were analyzed by Pearson correlation; otherwise, the data were compared by the Mann–Whitney test or Kruskal–Wallis test, and the relationship was analyzed by Spearman correlation. Statistical differences were considered significant at *p* < 0.05 and extremely significant at *p* < 0.01.

## Results

### Chemical Analysis of Bruceae Fructus Oil

**FIGURE 1:**
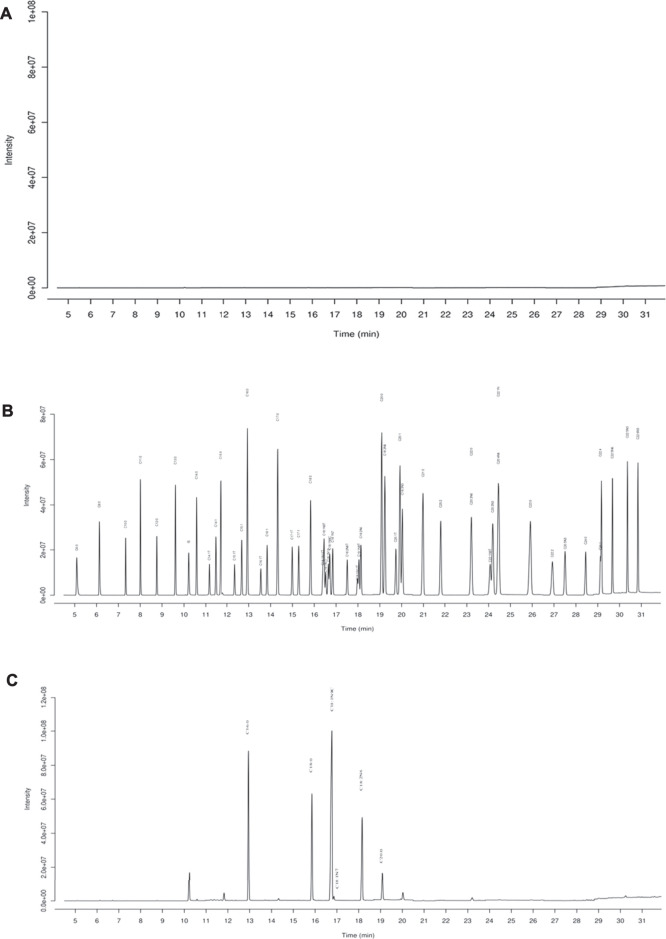
GC-MS chromatography for chemical analysis of BO. **(A)** Blank, **(B)** standards of fatty acid, **(C)** BO. C16:0 (12.94 min), palmitate; C18:0 (15.83 min), stearate; C18:1N9C (16.71 min), Oleate; C18:1N7 (16.84 min), vaccenate; C18:2N6 (18.14 min), linoleate; C20:0 (19.09 min), and arachidate.

GC-MS was employed to analyze the composition of BO quantitatively (Figure 1). A total of 46 fatty acids were quantified (Supplementary Tables S1–S3), and top 6 of them (>1,000 μg/ml) are listed in Table 1. It was found that the most abundant fatty acids, including vaccenate, oleate, linoleate, and palmitate, were consistent with the previous report (Tian et al., 2016).

**TABLE 1:** Top 6 fatty acids in BO (mean ± SD, *n* = 3).

Name	Abbreviation	Retention time (min)	Concentration (μg/ml)
Palmitate	C16:0	12.94	7,358.05 ± 289.55
Stearate	C18:0	15.83	5,155.03 ± 826.27
Oleate	C18:1N9C	16.71	13,891.98 ± 2,846.72
Vaccenate	C18:1N7	16.84	17,547.85 ± 3,191.5
Linoleate	C18:2N6	18.14	9,122.81 ± 83.59
Arachidate	C20:0	19.09	1,648.59 ± 166.87

### Bruceae Fructus Oil Has no Cytotoxicity on Breast Cancer Cell Lines *in vitro*

**FIGURE 2:**
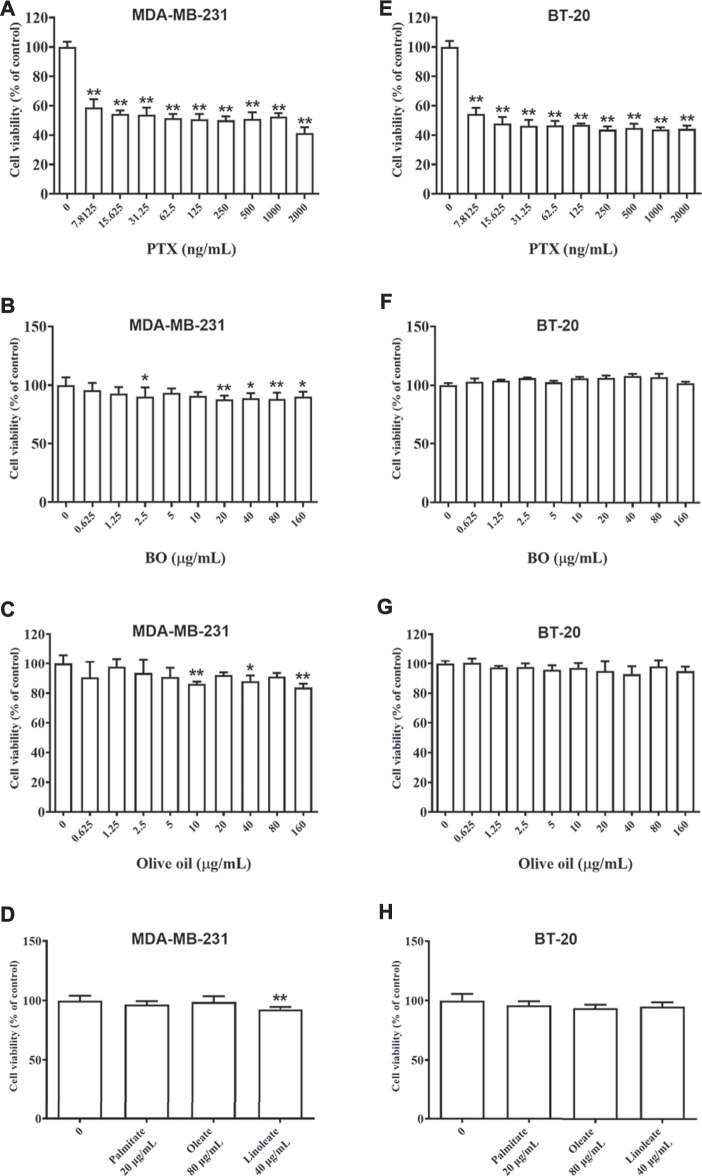
Cell viabilities of MDA-MB-231 **(A–D)** and BT-20 **(E–H**) are reduced by patclitaxel (PTX) but not affected by BO, olive oil, or the fatty acids (oleate, linoleate, palmitate). *n* = 5. *p < 0.05 and **p < 0.01.

As shown in Figure 2, PTX showed strong cytotoxicity on MDA-MB-231 cells and BT-20 cells, and the cell viability was merely about 60% even at the lowest dose of PTX (7.81 ng/ml, Figures 2A,E). After being exposed to BO (0.625–160 μg/ml) for 48 h, the cell viability of MDA-MB-231 cells of each concentration group was still all higher than 80%, although that of 40, 80, and 160 μg/ml displayed a statistical reduction (*p* < 0.01, Figure 2B). Similarly, BO showed no cytotoxicity on BT-20 *in vitro* (Figure 2F).

Olive oil is an edible vegetable oil, and it was found that its composition is similar to that of BO (Tian et al., 2016), including oleate, linoleate, and palmitate. Hence, the impact of olive oil on these two cell lines, as well as that of the three fatty acids, were also compared by the 48-h treatment at various concentrations. Data showed that neither olive oil nor any of the fatty acids had evident cytotoxicity on the two cell lines (Figures 2C,D,G,H).

### Bruceae Fructus Oil Exhibits a Gut Microbiota-dependent Tumor Suppression Without Toxicity in the Non-Targeted Organs

**FIGURE 3:**
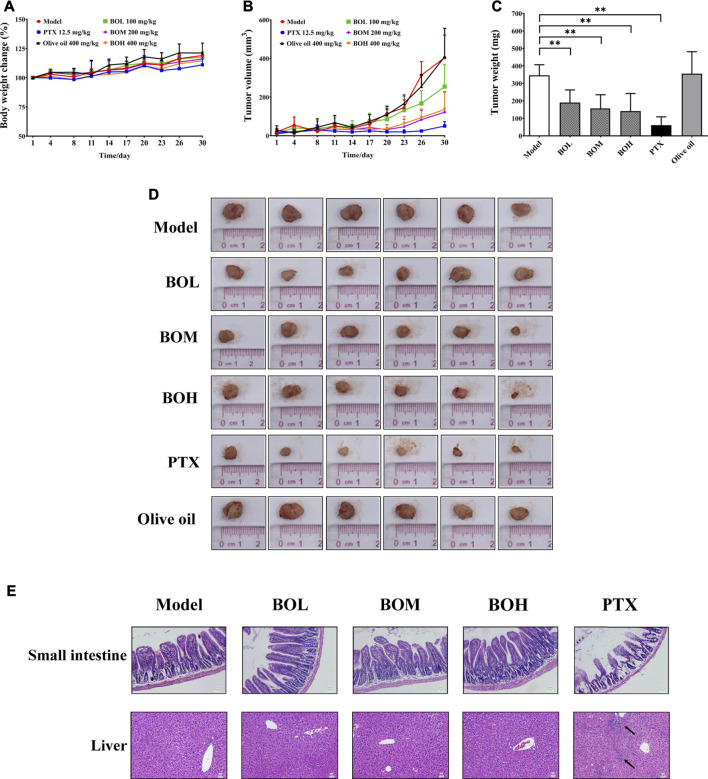
BO suppresses MDA-MB-231 tumor growth *in vivo* **(A–D)** but has no toxicity on the small intestine and liver (E). **(A)** Body weight change. **(B)** Tumor volume. **(C)** Tumor weight. **(D)** Tumor pictures. **(E)** Representative pictures of H&E staining at 200 × magnification, and the black arrows indicated the inflammatory infiltration. *n* = 6. *p < 0.05 and **p < 0.01.

The MDA-MB-231-xenograft murine breast cancer model was employed to investigate the tumor suppression potential of BO (Figures 3A–D). Body weights of all subjected mice were not affected by any of the treatment. The MDA-MB-231 tumor growth was significantly inhibited by PTX. Interestingly, BO, but not olive oil, effectively suppressed MDA-MB-231 tumor growth, although neither of them showed cytotoxicity *in vitro*. Moreover, the anticancer activity of BO was not accompanied by side effects in the non-targeting organs (Figure 3E). Although PTX evidently suppressed the tumor growth and it did not affect body weight, it induced apparent lesions in the small intestine, such as the shortened and atrophied villi, and the fractioned and incomplete muscular layer. PTX treatment also induced evident inflammatory infiltration in the liver. In contrast, BO did not induce lesions or inflammatory infiltration in these organs.

**FIGURE 4:**
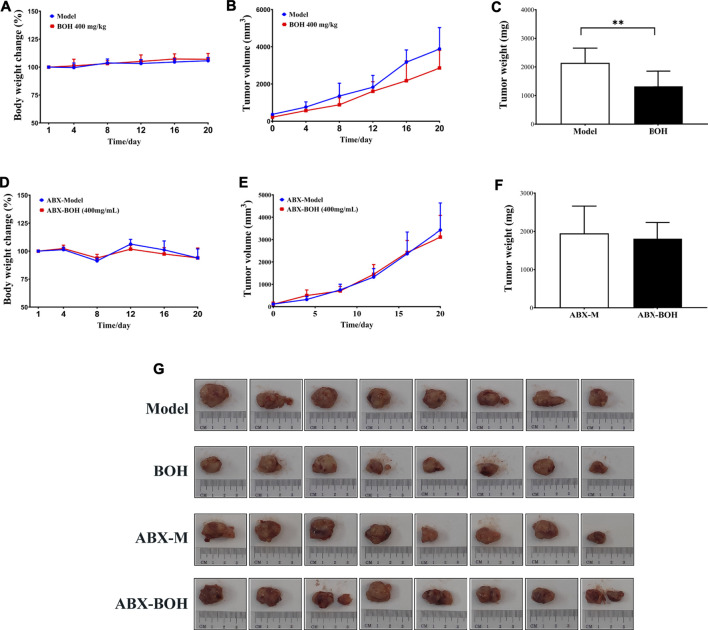
BO suppresses MDA-MB-231 tumor growth under SPF condition but failed under PSF condition. **(A–C)** Tumor volume and tumor weight under SPF condition. **(D–F)** Tumor volume and tumor weight under PGF condition. **(G)** Tumor pictures. *n* = 8. *p < 0.05 and **p < 0.01.

It is noteworthy that BO exhibited tumor suppression by oral administration but had no cytotoxicity on MDA-MB-231 cells, indicating the indispensable role of gut microbiota in the anti-tumor activity of BO. Hence, the impact of BO on tumor growth was compared in tumor-bearing mice under SPF condition and that under PGF condition. In this part, it was found that under SPF condition, BO (400 mg/kg, the BOH group) also evidently inhibited the tumor growth (*p* < 0.05, Figures 4A–C); while under PGF condition, BO (400 mg/kg, the ABX-BOH group) exhibited no inhibition on the tumor growth (*p* > 0.05, Figures 4D–F), suggesting that the tumor suppression by BO depends on gut microbiota.

### Bruceae Fructus Oil Regulates the Amino Acid Profile by Altering the Metabolism Function of Gut Microbiota

**FIGURE 5:**
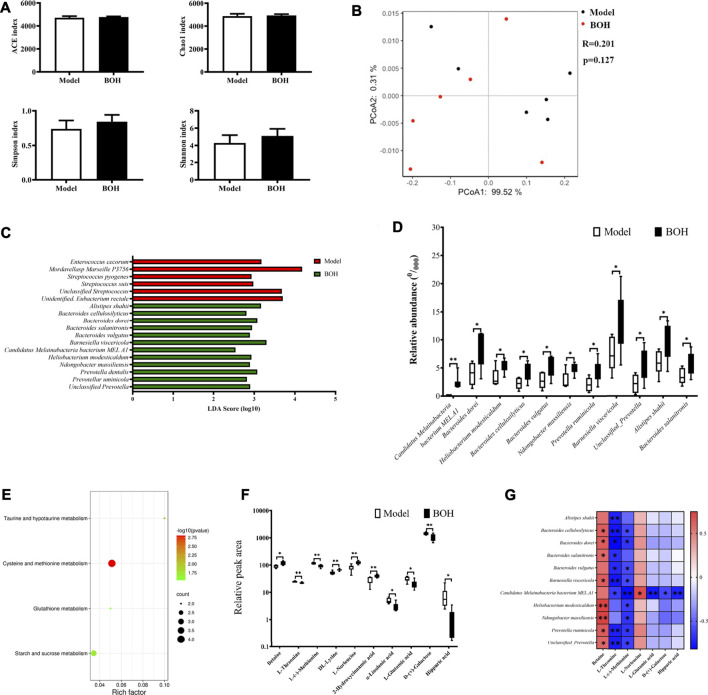
BO alters the taxonomy and function of gut microbiota and regulated amino acid profile in serum. **(A)** Indices of *a*-diversity analysis, including ACE, Chao1, Shannon and Simpson. **(B)** *ß*-diversity analysis, including UniFrac distance-based principal coordinates analysis (PCoA) and analysis of similarities (ANOSIM). **(C,D)** LEfSe analysis and the differential species. **(E)** Enriched function pathway based on differential genes. **(F,G)** Differential metabolites in serum and their correlations with the differential species. *n* = 6. *p < 0.05 and **p < 0.01 *vs.* model group.

As has been proved that gut microbiota played a critical role in the tumor suppression by BO, the regulation of BO on gut microbiota in the cecal content was analyzed by metagenome sequencing. The overall community structure was compared by α-diversity and β-diversity analyses. However, neither analyses found the difference between the two groups, suggesting that BO had no impact on the community structure of microbiota (Figures 5A,B). Although no diversity alternation was found, LEfSe analysis showed that there existed several specific species in each group (Figures 5C,D). There were 6 dominant species in the samples of the model group, that is, *Enterococcus cecorum*, *Streptococcus pyogenes*, *Streptococcus suis*, unclassified *Streptococcus*, *Mordavellasp Marseille* P3756, and unidentified *Eubacterium rectale*. Eleven species were significantly enriched in the samples of the BOH group. Especially, the relative abundances of *Candidatus Melainabacteria* bacterium *MEL.A1*, *Ndongobacter massiliensis*, *Prevotella ruminicola*, and an unclassified *Prevotella* in the BOH group were significantly increased to twice as those of the model group. Moreover, signaling pathway enrichment according to KEGG suggested that BO treatment have marked impacts on the following pathways: cysteine and methionine metabolism, taurine and hypotaurine metabolism, glutathione metabolism, and starch and sucrose metabolism (Figure 5E).

It is noteworthy that in the metabolism pathway of gut microbiota, several amino acid metabolism pathways were evidently impacted by BO treatment. Therefore, untargeted metabolomics by UPLC-MS/MS was applied to explore the effect of BO on the metabolite profile of serum. It was found that BO treatment remarkably increased betaine, DL-lysine, L-norleucine, and 2-hydroxycinnamic acid, while it decreased L-threonine, L-(-)-methionine, hippuric acid, *a*-linolenic acid, L-glutamic acid, and D-(+)-galactose (Figure 5F). And there were notable correlations between changes of metabolites and those of microorganisms (Figure 5G). In terms of metabolites, L-(-)-methionine and L-threonine were significantly negatively related to most of the species enriched by BO (*r* = −0.8∼−0.4, *p* < 0.05 or 0.01), while betaine had positive correlations with most of them (*r* = 0.5–0.8, *p* < 0.05 or 0.01); in terms of microorganisms, the relative abundance of *Candidatus Melainabacteria bacterium MEL.A1* was negatively related to L-threonine, L-(-)-methionine, L-glutamic acid, D-(+)-galactose, and hippuric acid (*r* = −0.8∼−0.6, *p* < 0.05 or 0.01) but positively to L-norleucine (*r* = 0.70, *p* < 0.05). It is noteworthy that the significant changes in metabolites in serum were involved in several amino acid metabolism, that is, L-(−)-methionine in “cysteine and methionine metabolism,” L-glutamate in “taurine and hypotaurine metabolism,” and “glutathione metabolism,” which was in accordance with the impact on the amino acid metabolism function of the gut microbiota itself. All these implied that BO would regulate the host’s amino acid profile by altering the metabolism function of gut microbiota, thus contributing to the gut microbiota-dependent tumor suppression.

### Bruceae Fructus Oil Reduces Tumor Burden by Restraining Autophagy *via* mTOR Activation

**FIGURE 6:**
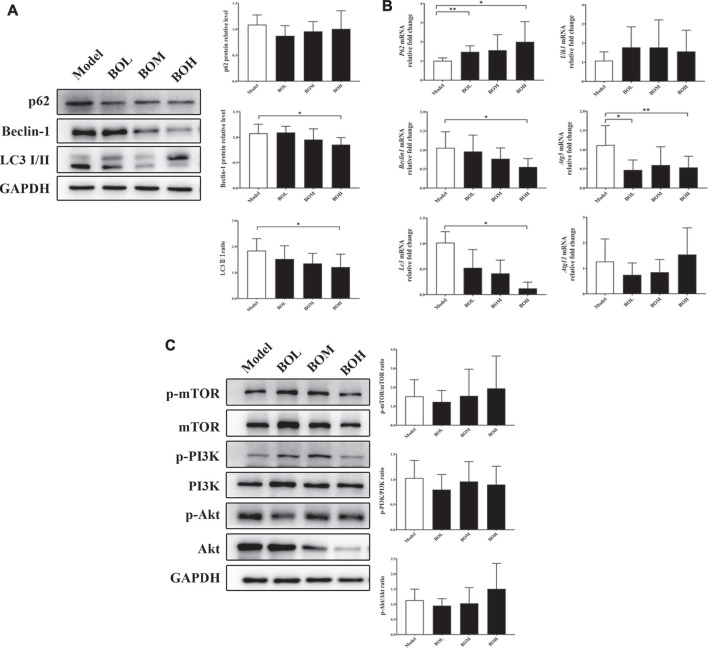
BO restrains autophagy in MDA-MB-231 tumor without impact on PI3k/Akt/mTOR axis. **(A)** Protein expressions of autophagy-related proteins. **(B)** mRNA levels of autophagy-related genes. **(C)** Phosphorylations of mTOR, PI3K, and Akt. *n* = 6. *p < 0.05 and **p < 0.01 *vs.* model group.

**FIGURE 7:**
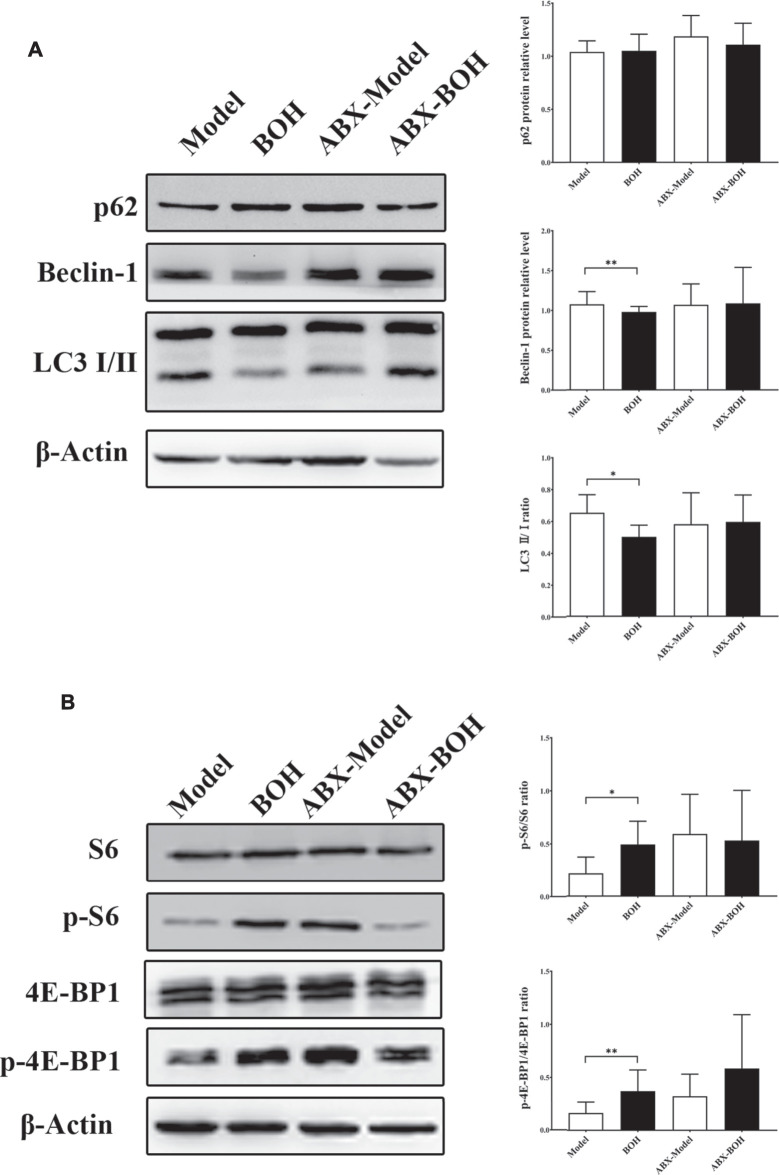
BO activates mTOR as indicated by the phosphorylation of S6 and 4E-BP1. **(A)** Expressions of autophagy-related proteins under SPF condition and PGF condition. **(B)** Phosphorylation of S6 and 4E-BP1. *n* = 6. *p < 0.05 and **p < 0.01.

Given that MDA-MB-231 is a TNBC cell line with hyper-autophagy (Maycotte et al., 2014; Garbar et al., 2017), we explored the possible involvement of autophagy in the inhibition by BO. Results showed that BO significantly decreased the ratio of LC 3 II/I and the protein expression of beclin-1 (Figure 6A). BO upregulated the mRNA level of *p62*, while downregulated that of *beclin1*, *Atg5*, and *Lc3* (Figure 6B). Compared with the effect under SPF condition, it was found that BO failed to decrease the ratio of LC 3 II/I and the expression of beclin-1 under PGF condition, indicating that microbiota depletion makes the BO’s autophagy restrain wear off (Figure 7A).

The mammalian target of rapamycin (mTOR) is a crucial sensor for extracellular nutrients in cell physiological activity, especially as an upstream inhibitor of autophagy. Ribosomal protein S6 and 4E-binding protein 1 (4E-BP1) are the downstream proteins of mTOR. It has been proved that the PI3K/Akt/mTOR axis and amino acid stimulation are the two ways of mTOR activation, and phosphorylation of the downstream (such as S6 and 4E-BP1) would serve as an indicator for the kinase activity of mTOR (Shi et al., 2019). Our data showed that although not changing the phosphorylation of PI3K, Akt, or mTOR (Figure 6C), BO promoted phosphorylation of S6 and 4E-BP1 under SPF condition, while such promotion disappeared under PGF condition (Figure 7B). The data indicated that autophagy restraint contributed to the tumor suppression by BO, which is possibly mediated by the mTOR activation *via* amino acid metabolism regulation.

## Discussion

The anticancer potential of *B. javanica* has been investigated since as early as 1980s. It was found that bruceantin could have been available in breast cancer treatment owing to the outstanding cytotoxicity, but its clinical trial was aborted at phase II due to the severe side effects (Wiseman et al., 1982; Arseneau et al., 1983). By contrast, BO has been approved and developed as a marketable drug with various dosage forms, such as oral emulsion, parenteral emulsion, and soft capsules. Nowadays, BO preparations are adjunctively applied in treatment for gastrointestinal cancer, lung cancer, and brain metastasis of lung cancer, while the effect of BO on breast cancer has not yet been explored. It has been revealed that BO and various cooking oils (such as olive oil, coix seed oil, sunflower seed oil, and peanut oil) have similar composition of fatty acids, which are mainly oleate, linoleate, and palmitate (Tian et al., 2016). In the present study, it was found that BO, olive oil, or the fatty acids (oleate, linoleate, and palmitate), had no direct cytotoxicity on the TNBC cell lines MDA-MB-231 or BT-20 *in vitro*. Nevertheless, it is quite surprising that oral administration of BO evidently suppressed the tumor growth *in vivo*, while olive oil did not have any impact on tumor growth. Moreover, the tumor inhibition of BO was not accompanied by side effects in the small intestine or liver. These results indicate that BO would be a promising candidate for novel therapy against TNCB with notable efficacy and safety.

Numerous studies have revealed the pivotal role of gut microbiota for cancer therapy responses (Baruch et al., 2020; Guo et al., 2020). Given that BO exhibited tumor suppression only by oral administration, we compared the effect of BO in tumor-bearing mice under SPF condition and that under PGF condition. It is a well-established model that applies broad-spectrum antibiotics by oral administration, including ampicillin, metronidazole, neomycin, and vancomycin, to deplete gut microorganisms to verify the necessity of the gut microbiota for drug efficacy (Gopalakrishnan et al., 2018; Routy et al., 2018). As expected, BO was not able to suppress tumor growth under PGF when compared with that under SPF condition, in which the mice were raised in gnotobiotic facility and orally treated with broad-spectrum antibiotics prior to tumor induction and BO treatment. This result suggested that the gut microbiota is indispensable for the tumor inhibition of BO. Therefore, metagenome sequencing and untargeted metabolomics were employed to explore the details about the role of microbiota regulation in BO’s tumor suppression.

It was found that BO evidently altered the microorganism composition and its metabolism function. Although the community diversity was not affected, several species were enriched by BO treatment, and the relative abundances of *Candidatus Melainabacteria bacterium MEL.A1*, *Ndongobacter massiliensis*, *Prevotella ruminicola* and an unclassified *Prevotella* were even significantly increased to twice. Metabolism functions were also remarkably modulated by BO in addition to the impact on species amount. It was found that BO obviously regulated several amino acid metabolism pathways in gut microbiota, including cysteine and methionine metabolism, taurine and hypotaurine metabolism, and glutathione metabolism. Given that the host’s metabolism would be interacted with the gut microbiota, we employed untargeted metabolomics by UPLC-MS/MS to explore the impact of BO on serum metabolite profiles. Data showed that BO evidently regulated the relative amount of several amino acids, including lysine, leucine, threonine, and methionine. Interestingly, these regulated amino acids not only displayed notable correlations with the enriched microorganisms by BO but also involved in the modulated amino acid metabolism pathway of the gut microbiota itself, that is, L-(−)-methionine in “cysteine and methionine metabolism,” L-glutamate in “taurine and hypotaurine metabolism,” and “glutathione metabolism.” Amino acids are the key cornerstones for all vital activity, and they also have crucial roles in the development of cancer. Certain tumor cells become auxotrophic for specific amino acids, such as asparagine, arginine, and methionine, which has attracted increasing attention for novel treatment strategy by employing amino acid–degrading enzymes (Wang et al., 2020). Methionine is an essential amino acid found in foods, and it is involved in various important processes, such as in the methylation of various cellular components and in the formation of polyamines for cell division and proliferation (Cellarier et al., 2003). Of note, the methionine dependence of certain cancer cells has been revealed as early as in the 1950s (Sugimura et al., 1959; Halpern et al., 1974), but it is often a selective vulnerability due to heterogeneity. It has been found that methionine restriction would activate the integrated stress response in TNBC cells (Rajanala et al., 2019) and block the tumor initiation of lung cancer (Wang Z. et al., 2019). Taken together, BO exhibits a promising tumor suppression that is contributed by the regulation on the host’s amino acid metabolism, which depends on the metabolism alternation of gut microbiota.

Autophagy is one of the critical interventions by amino acids in cells, which is a lysosomal degradation pathway for nutrient recycling, including amino acids, sugars, and lipids (Noguchi et al., 2020). Indeed, autophagy has been proposed as a “double-edged sword” for cancer development. In the initial stage of formation, the host itself would employ autophagy as a mechanism of tumor suppression to reduce proteins and structural substrates for cell proliferation (Lin and Baehrecke, 2015). However in the advanced stages, autophagy is the most optimal approach to endow cancer cells with metabolic flexibility, which enables the cancer cells to recycle their own nutrients for the survival in nutrient-limiting and hypoxia tumor microenvironments (TMEs) (Rabinowitz and White, 2010). Recent studies demonstrated that due to the substantially higher number of autophagosomes, the basal autophagy of TNBC, such as MDA-MB-231 cell line, was higher than that of the other types of breast cancer (Maycotte et al., 2014; Garbar et al., 2017), suggesting that active autophagy is indispensable for the proliferation and survival of TNBC. Our results showed that under SPF condition, BO treatment decreased the ratio of LC3 II/I, indicating the lipid conjugation of LC3, a well-established autophagosome marker (Klionsky et al., 2016), was suppressed. Moreover, BO downregulated the expressions of beclin-1, as well as several mRNA levels of autophagy-related genes (*Beclin1*, *Atg5* and *Lc3*), suggesting that the inhibition on autophagy plays a critical role in tumor suppression by BO.

Autophagy responses to nutrient-limiting and hypoxia stress *via* the negative regulation of mTOR (Kim and Guan, 2019). Indeed, mTOR is an atypical serine/threonine kinase, and functionally, it is a master growth regulator that senses and integrates diverse nutritional and environmental cues, including growth factors, energy levels, cellular stress, and amino acids. The growth factor–mediated PI3K/Akt/mTOR axis is a frequently activated pathway in cancer cells due to AKT1 mutation, loss of PTEN, or PI3K activator mutation (du Rusquec et al., 2020). In contrast, amino acids’ signal to mTOR is independent from the PI3K/Akt/mTOR axis. Both pathways enable mTOR to carry out kinase activity by directly phosphorylating 4E-BP1 and ribosomal protein S6 kinase 1 (p70S6k), thus initiating the eukaryotic translation *via* phosphorylations of S6 and eIF4 (Follo et al., 2019; Shi et al., 2019; Yoder et al., 2019; Iffland et al., 2020). We found that BO treatment when accompanied with the regulation on amino acid metabolism promoted the phosphorylation of S6 and 4E-BP1, rather than those of PI3K, Akt, or mTOR, indicating that mTOR was activated by BO in an amino acid–regulated form, which correspondingly restrained autophagy to suppress tumor growth. By contrast, the failure of tumor suppression by BO under PGF condition resulted in indistinctive changes in autophagy activity (expressions of the autophagy-related protein) and mTOR activity (phosphorylation of S6 and 4E-BP1). These findings, once again, verified the dependence of gut microbiota for the tumor inhibition of BO.

Recently, gut microbiota was revealed as a vital assistant in certain drug efficacy. For example, Wang and colleagues found that oral administration of berberine, but not injection, improved brain dopa/dopamine levels relying on the tyrosine hydroxylase of *Enterococcus* in gut microbiota (Wang Y. et al., 2021). Therefore, berberine is able to ameliorate Parkinson’s disease, although it is poorly absorbed in the intestine, which provides a credible answer for the controversial results about berberine’s efficacy (Kwon et al., 2010; Durairajan et al., 2012; Kim et al., 2014). In the present study, we found that BO efficiently suppressed tumor growth relying on the gut microbiota, while olive oil failed, and neither BO itself nor the constituents of it, including oleate, linoleate, and palmitate, had direct cytotoxicity on MDA-MB-231 or BT-20 cell lines. Undoubtedly, certain compounds of BO are responsible for its regulation on the gut microbiota, thus modulating the host’s metabolism to suppress tumor growth. Brusatol is one of the characteristic components of *B. javanica*, which has been found to be cytotoxic to cancer cells (Lee J. H. et al., 2020; Xie et al., 2021). However, it was found that there is a small amount of brusatol in BO (<0.001%) (Wang T. et al., 2021), which is far from the amount that is able to suppress tumor. It would be interesting and significative to explore whether brusatol, or a synergy of brusatol and other compounds (such as the most abundant fatty acids), is responsible for the regulation on microbiota, and it is also necessary to verify the target microorganism of BO, as well as the corresponding gene, to make comprehensive interpretation for its anti–breast cancer activity.

## Conclusion

**FIGURE 8:**
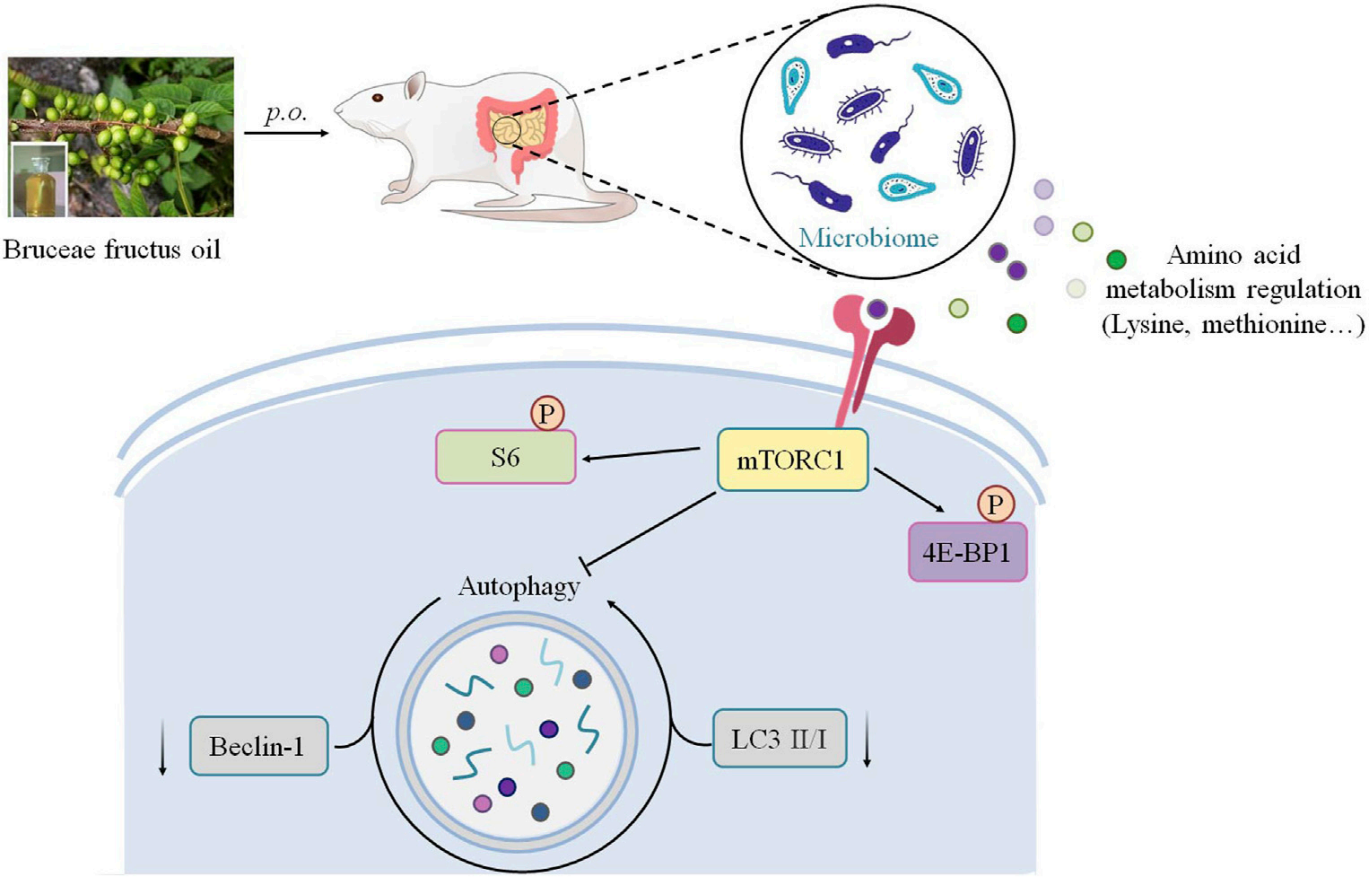
Schematic illustration depicting the potential mechanism associated with the gut microbiota–dependent tumor suppression of BO on TNBC.

Overall, this study revealed an anti–breast cancer potential of Bruceae fructus oil (BO). BO exhibited a gut microbiota–dependent tumor suppression without toxicity in the non-targeted organs. Mechanically, BO regulated the host’s amino acid profile *via* the metabolism alternation of the gut microbiota, and then mTOR was activated in an amino acid–regulated form to restrain the hyper-autophagy within the MDA-MB-231 tumor, resulting in a distinct tumor suppression (Figure 8). Although the exact constituents responsible for the anti–breast cancer activity of BO remain to be explored, the present study highlighted a promising application of BO against breast cancer with novel efficacy and safety.

## Data Availability

The data presented in the study are deposited in the NCBI repository, accession number PRJNA748454 (https://dataview.ncbi.nlm.nih.gov/object/PRJNA748454?reviewer=aisfmuhda17ttj0qsdrggm5iq8).

## Abbreviations

4EBP1, 4E-binding protein 1; ANOSIM, analysis of similarities; Atg, autophagy-related gene; BCA, bicinchoninic acid; BO, Bruceae fructus oil; LEfSe, linear discriminant analysis effect size; mTOR, mammalian target of rapamycin; MTT, 3-(4,5-dimethyl-2-thiazolyl)-2,5-diphenyl-2-H-tetrazolium bromide; PCoA, principal coordinate analysis; PGF, pseudo germ-free; PI3K, phosphoinositide 3-kinase; PTX, paclitaxel injection; S6, ribosomal protein S6; SPF, specific pathogen-free; TNBC, triple-negative breast cancer; UPLC-MS/MS, ultrahigh performance liquid chromatography–tandem mass spectroscopy.
