# Supplementary material for: Bruceae Fructus Oil Inhibits Triple-Negative Breast Cancer by Restraining Autophagy: Dependence on the Gut Microbiota-Mediated Amino Acid Regulation
Source: Front Pharmacol. 2021 Oct 1;12:727082. doi: 10.3389/fphar.2021.727082 (PMC8517338; doi:10.3389/fphar.2021.727082)
Supplement: Supplementary file 1 [file DataSheet1.zip › Supplementary materials and raw data/Raw data/Un-cropped WB images for Fig 6.pdf]

Fig. 6 A Beclin-1, and the reference protein.

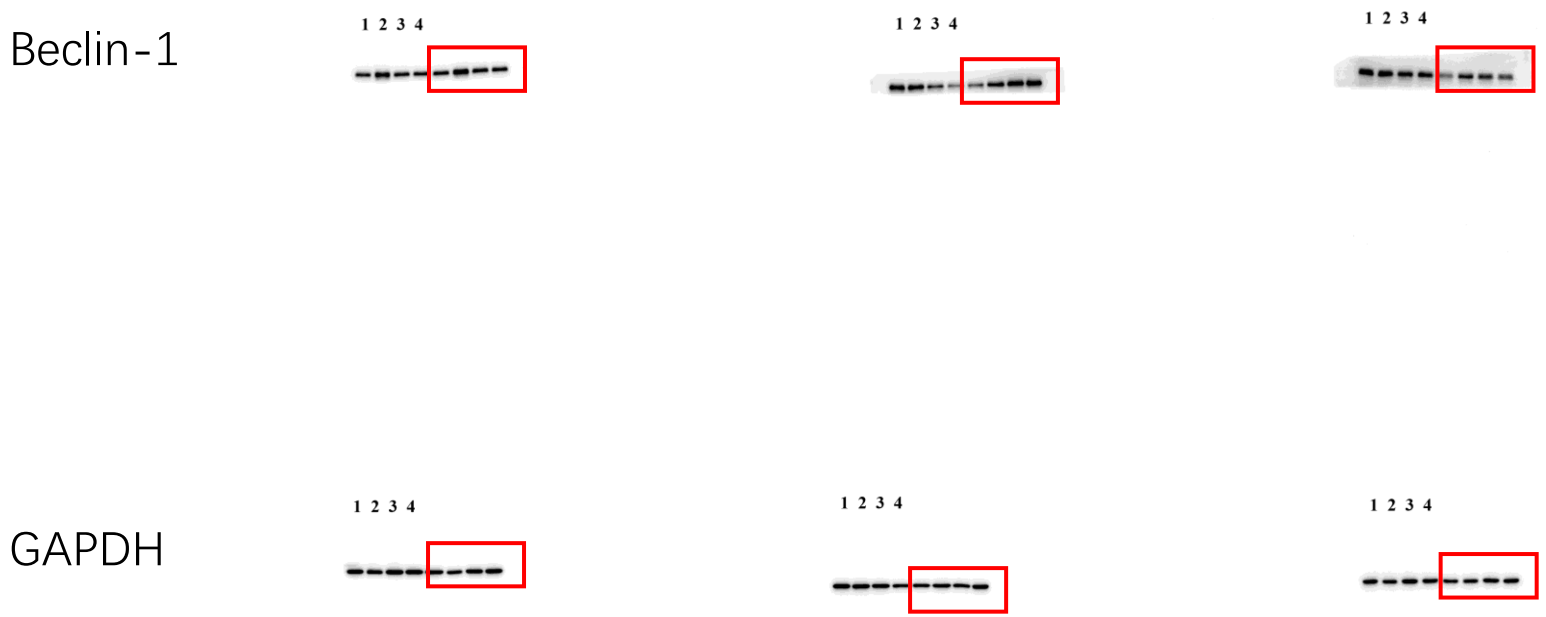

Lane 1: Model
Lane 2: BOL
Lane 3: BOM
Lane 4: BOH
Red frame： These blots were samples of a related project, which are not being used in this manuscript.

Fig. 6 A Beclin-1, and the reference protein.

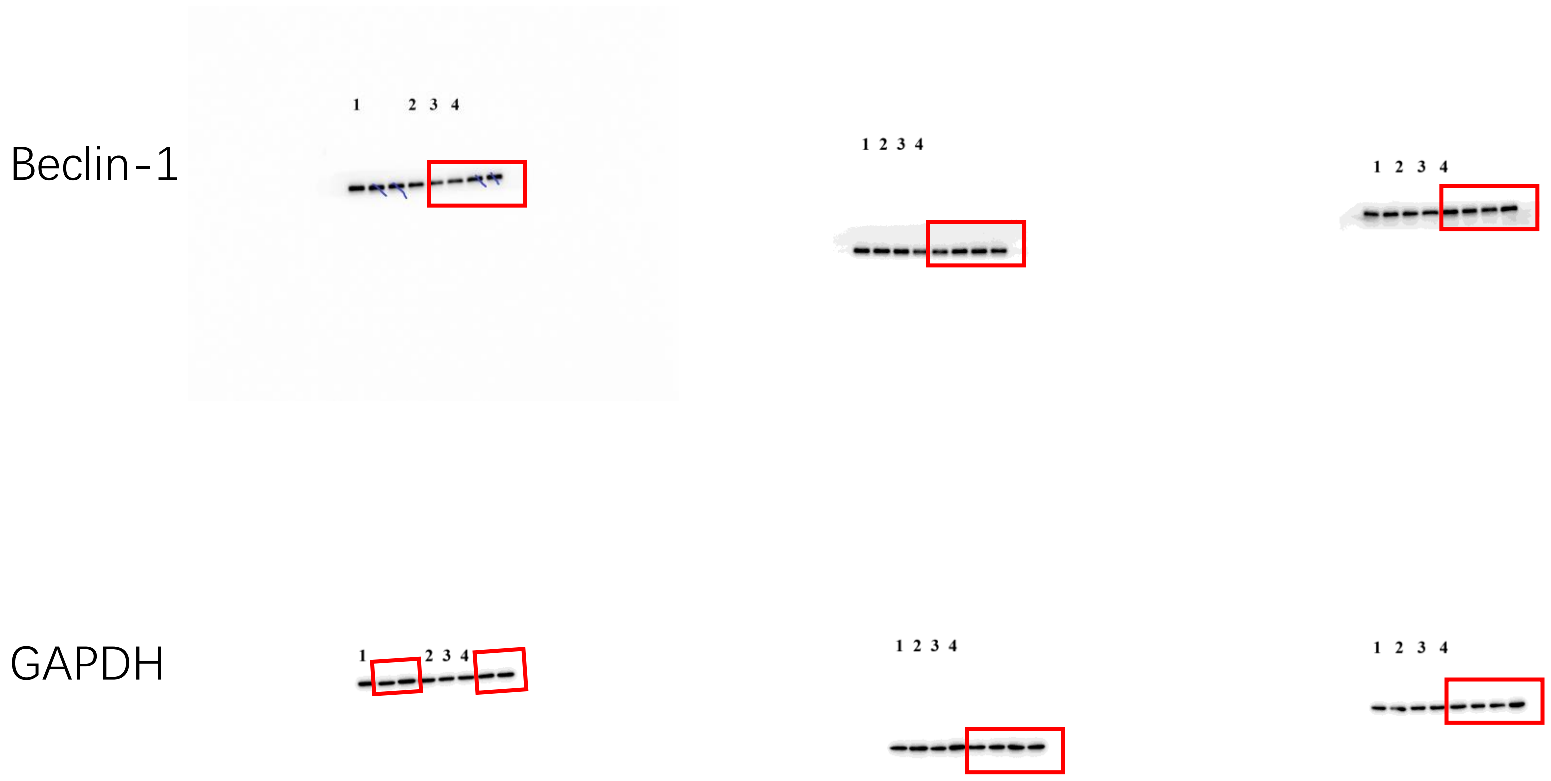

Lane 1: Model
Lane 2: BOL
Lane 3: BOM
Lane 4: BOH
Red frame： These blots were samples of a related project, which are not being used in this manuscript.

Fig. 6 A LC 3II/I, and the reference protein.

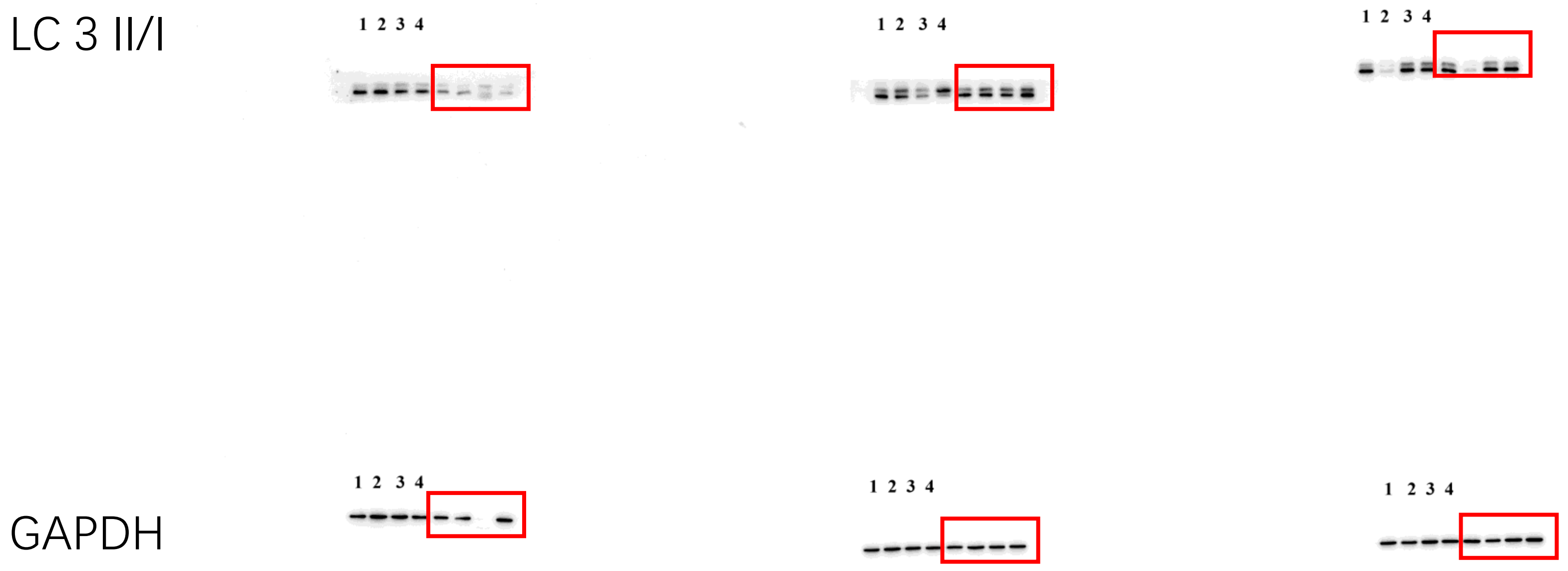

Lane 1: Model
Lane 2: BOL
Lane 3: BOM
Lane 4: BOH
Red frame： These blots were samples of a related project, which are not being used in this manuscript.

Fig. 6 A LC 3II/I, and the reference protein.

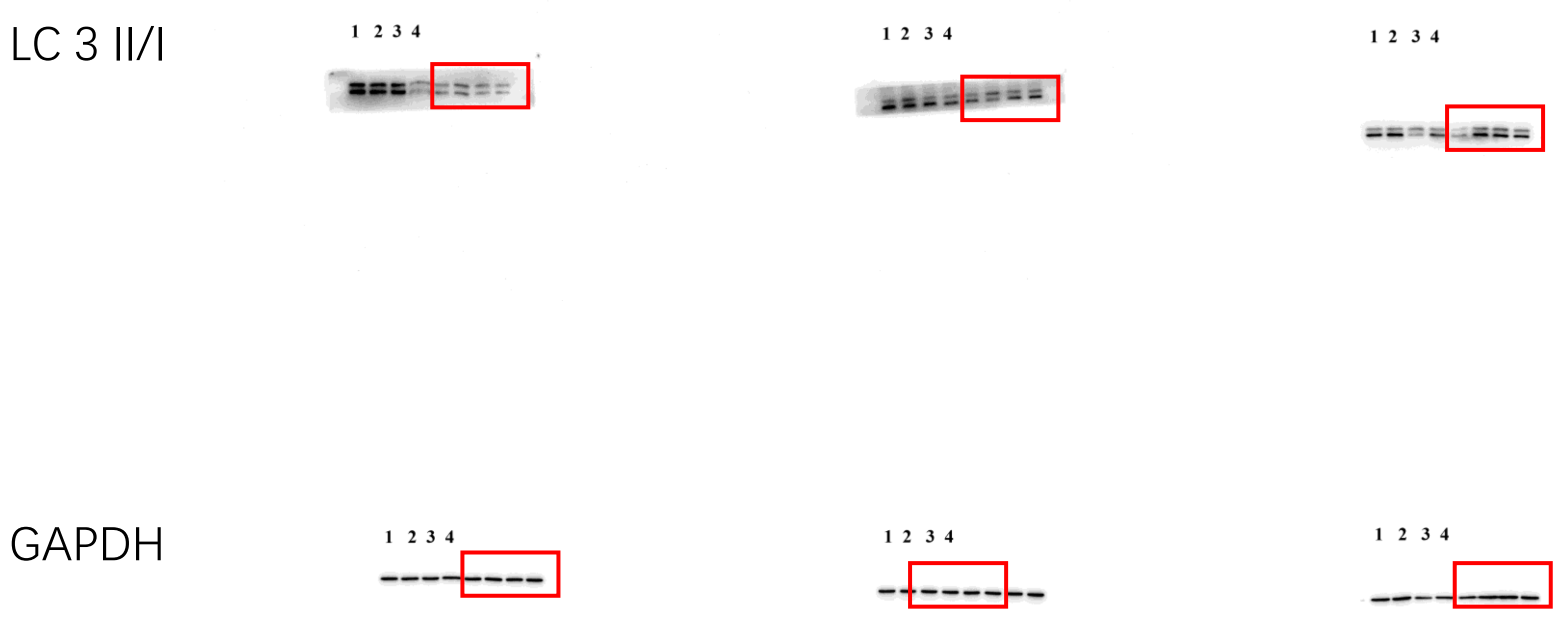

Lane 1: Model
Lane 2: BOL
Lane 3: BOM
Lane 4: BOH
Red frame： These blots were samples of a related project, which are not being used in this manuscript.

Fig. 6 A p62, and the reference protein.

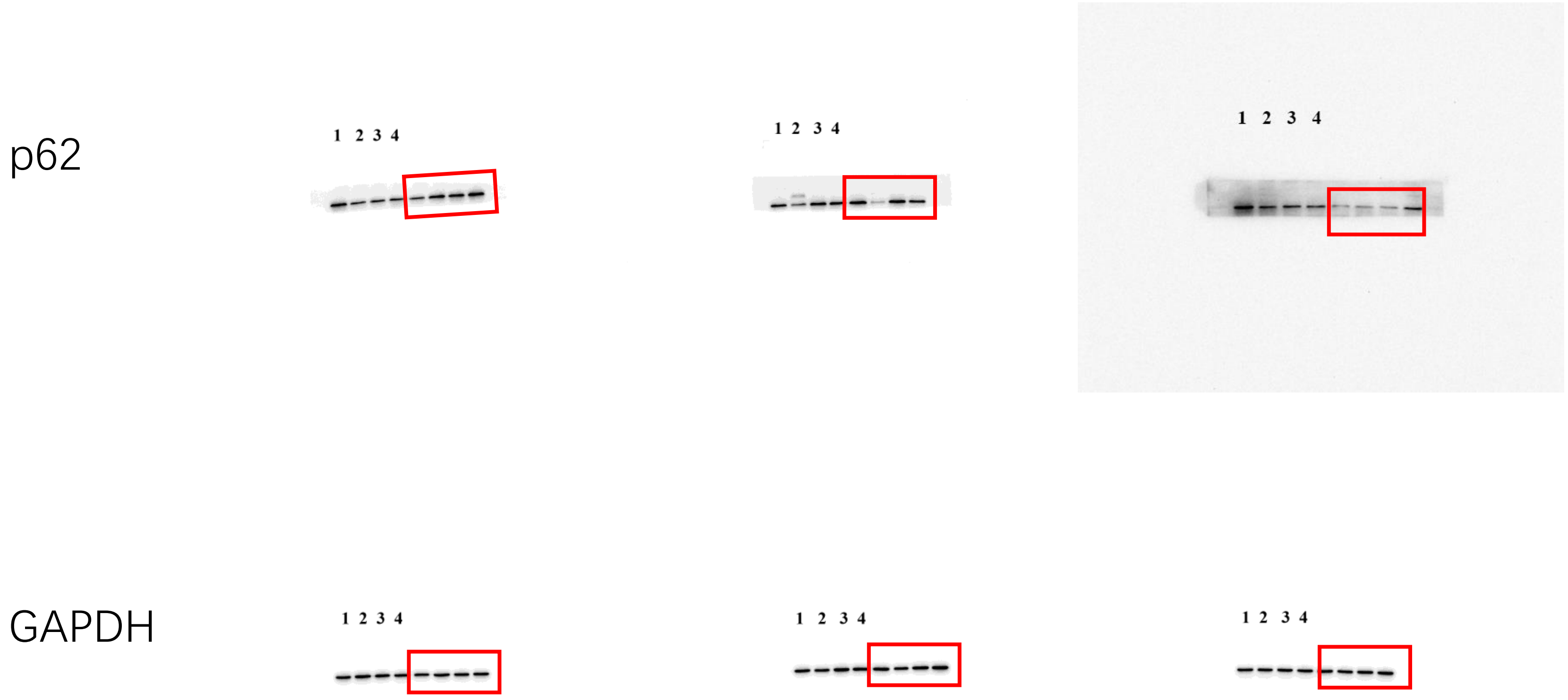

Lane 1: Model
Lane 2: BOL
Lane 3: BOM
Lane 4: BOH
Red frame： These blots were samples of a related project, which are not being used in this manuscript.

Fig. 6 A p62, and the reference protein.

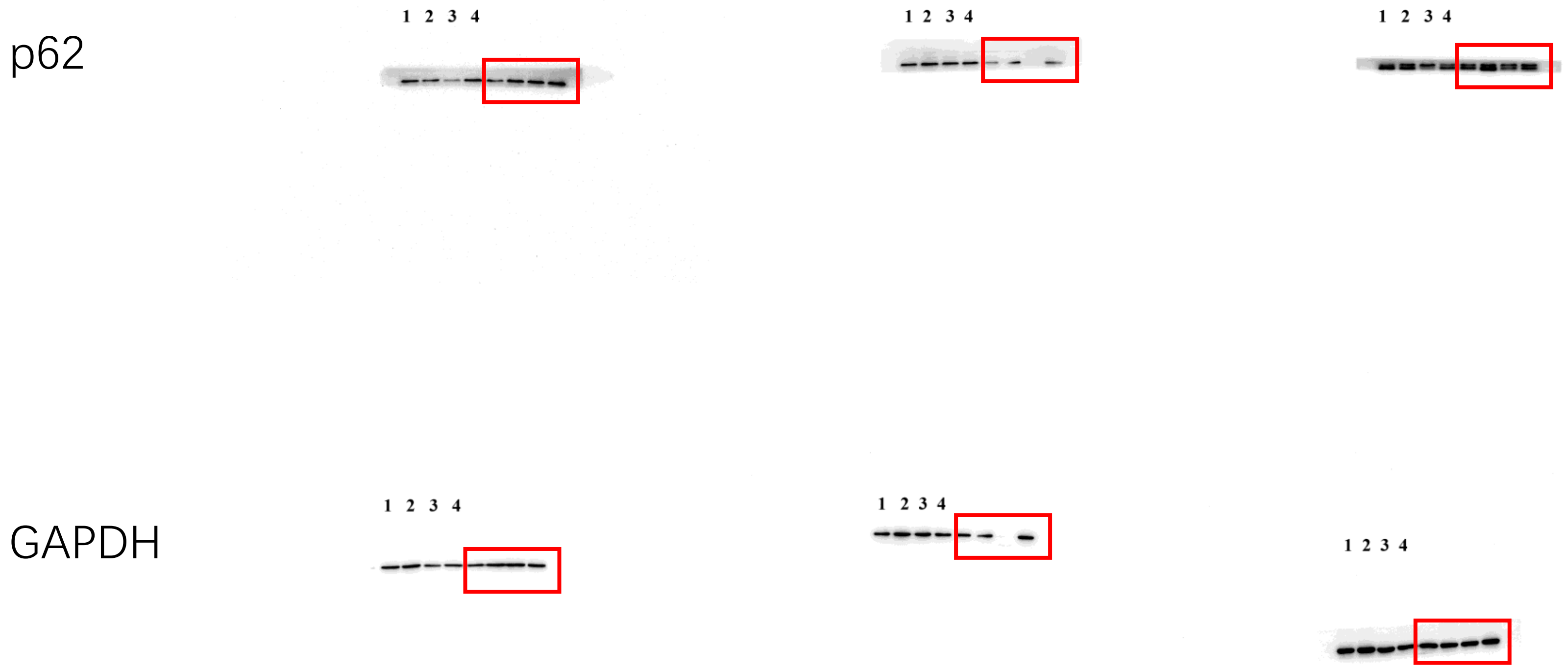

Lane 1: Model
Lane 2: BOL
Lane 3: BOM
Lane 4: BOH
Red frame: These blots were samples of a related project, which are not being used in this manuscript.

Fig. 6 C AKt, and the reference protein.

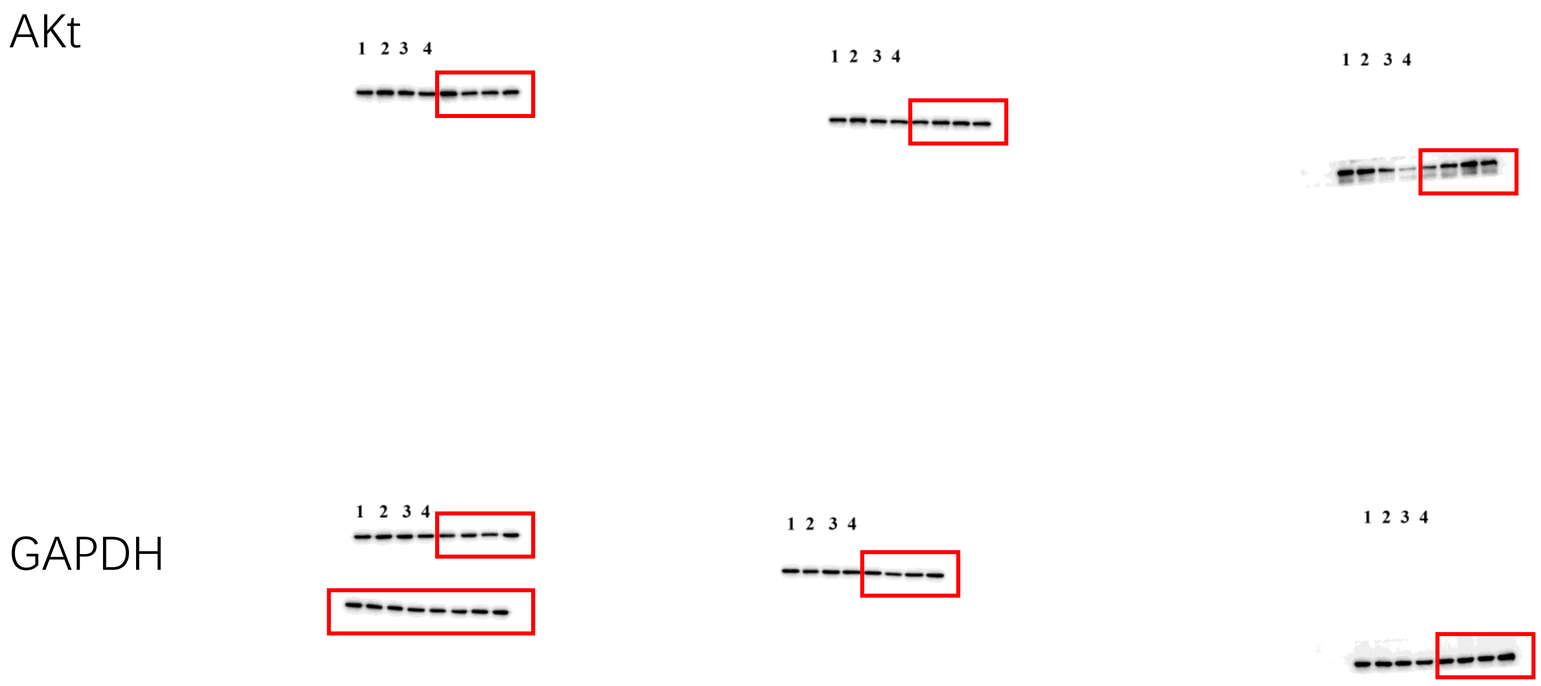

Lane 1: Model
Lane 2: BOL
Lane 3: BOM
Lane 4: BOH
Red frame： These blots were samples of a related project, which are not being used in this manuscript.

Fig. 6 C AKt, and the reference protein.

AKt

GAPDH

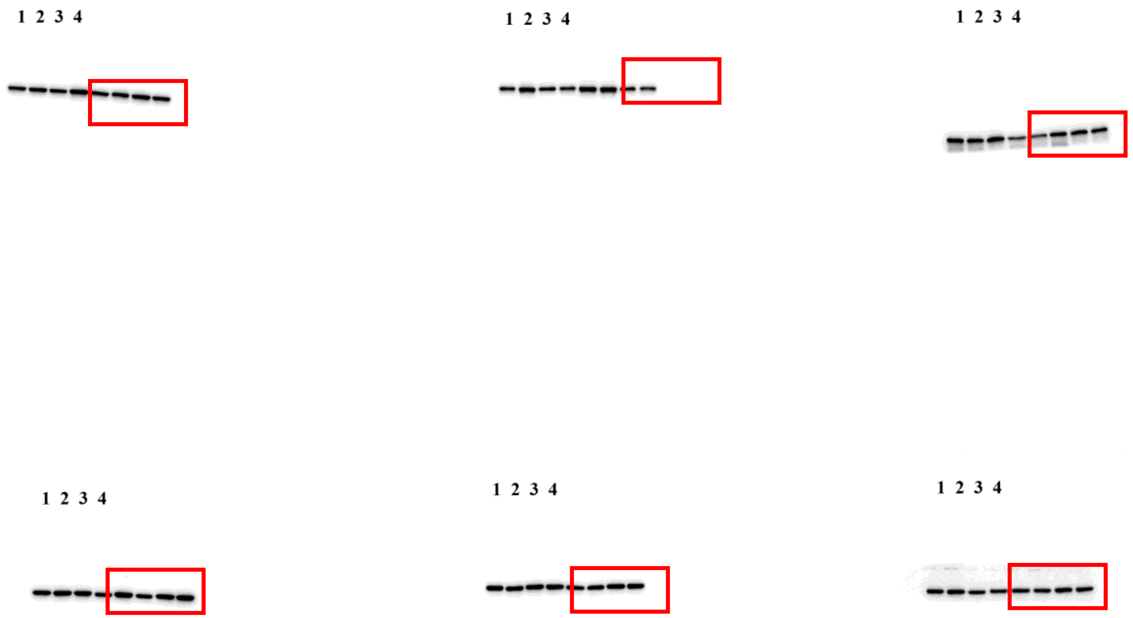

Lane 1: Model
Lane 2: BOL
Lane 3: BOM
Lane 4: BOH
Red frame： These blots were samples of a related project, which are not being used in this manuscript.

Fig. 6 C p-AKt, and the reference protein.

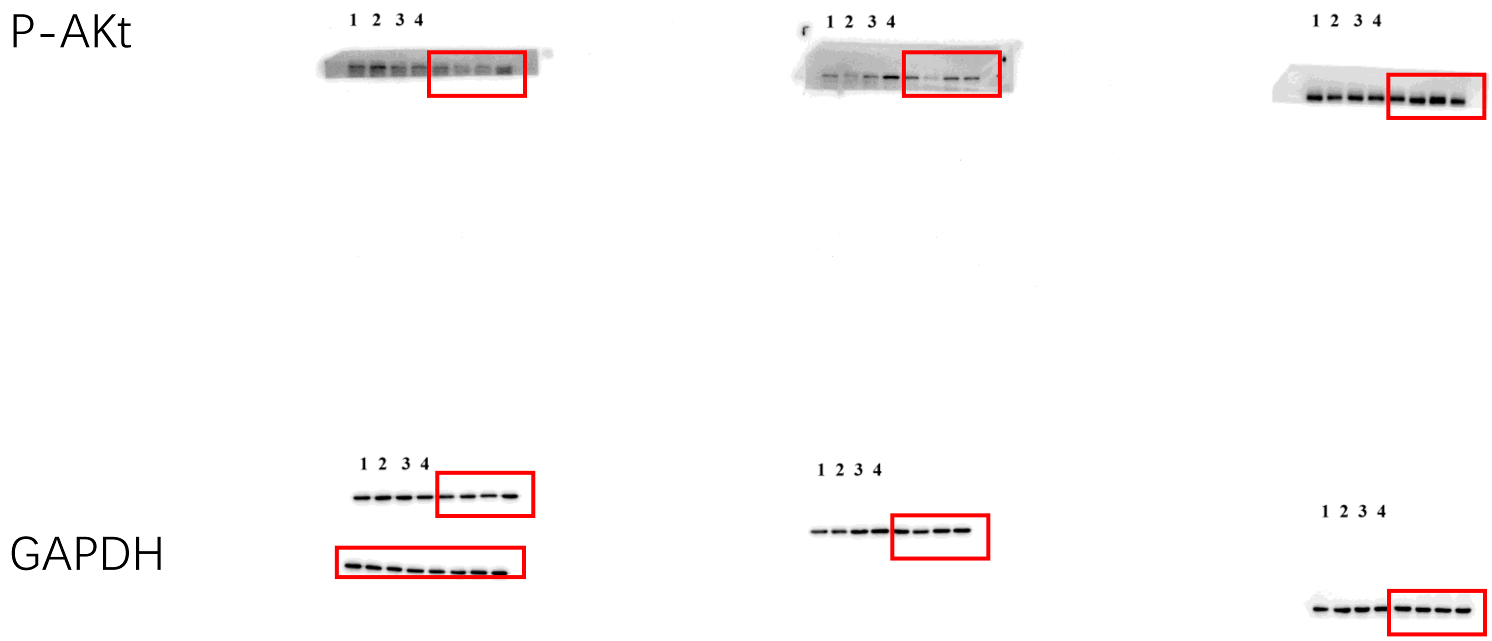

Lane 1: Model
Lane 2: BOL
Lane 3: BOM
Lane 4: BOH
Red frame： These blots were samples of a related project, which are not being used in this manuscript.

Fig. 6 C p-AKt, and the reference protein.

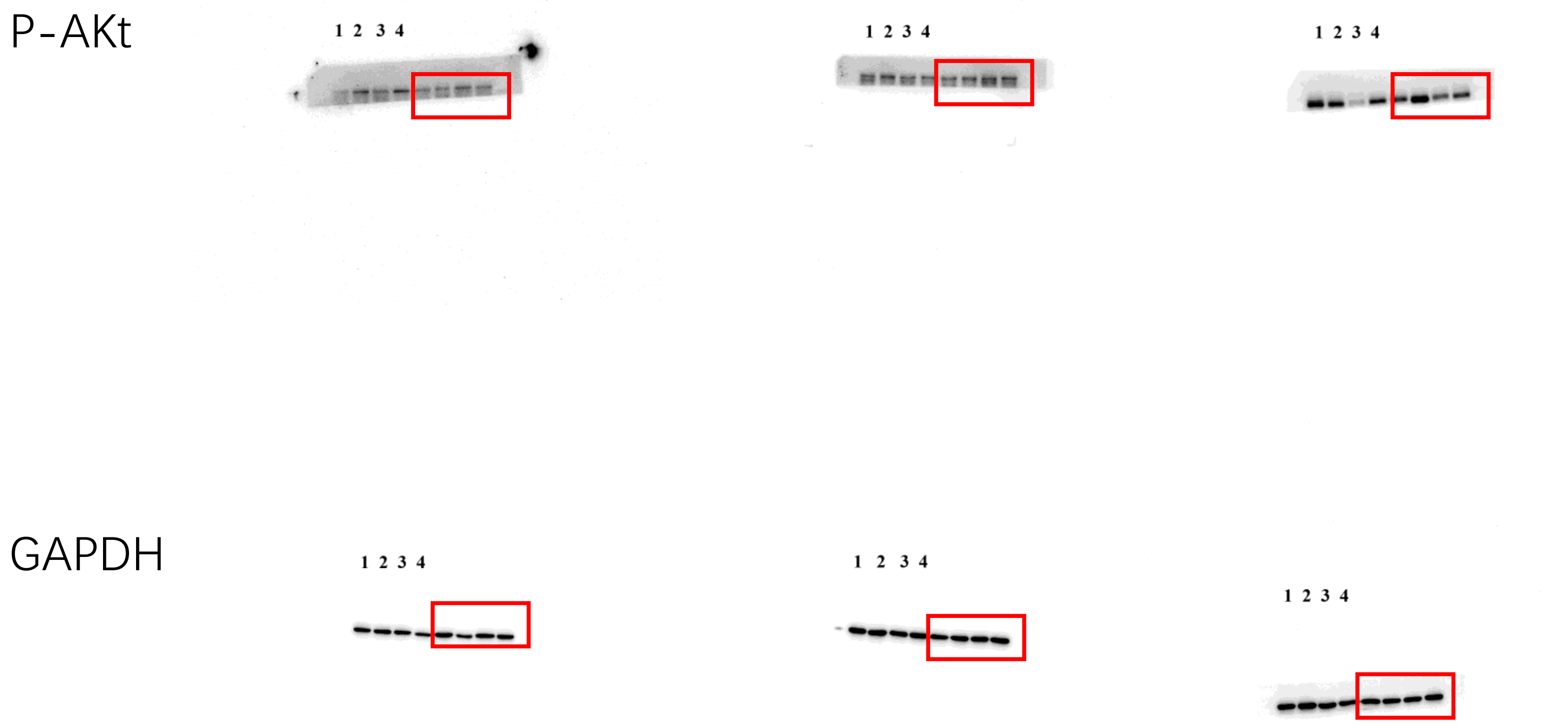

Lane 1: Model
Lane 2: BOL
Lane 3: BOM
Lane 4: BOH
Red frame: These blots were samples of a related project, which are not being used in this manuscript.

Fig. 6 C mTOR, and the reference protein.

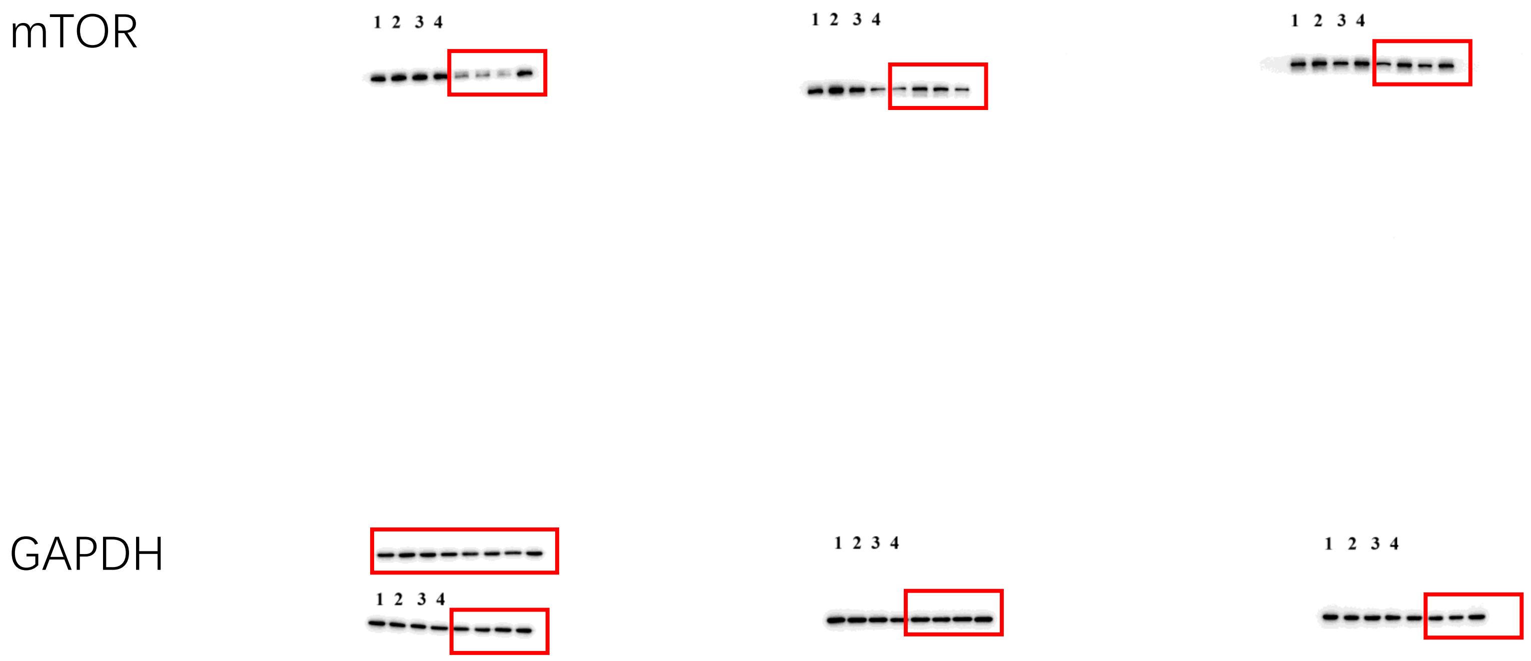

Lane 1: Model
Lane 2: BOL
Lane 3: BOM
Lane 4: BOH
Red frame： These blots were samples of a related project, which are not being used in this manuscript.

Fig. 6 C mTOR, and the reference protein.

mTOR

GAPDH

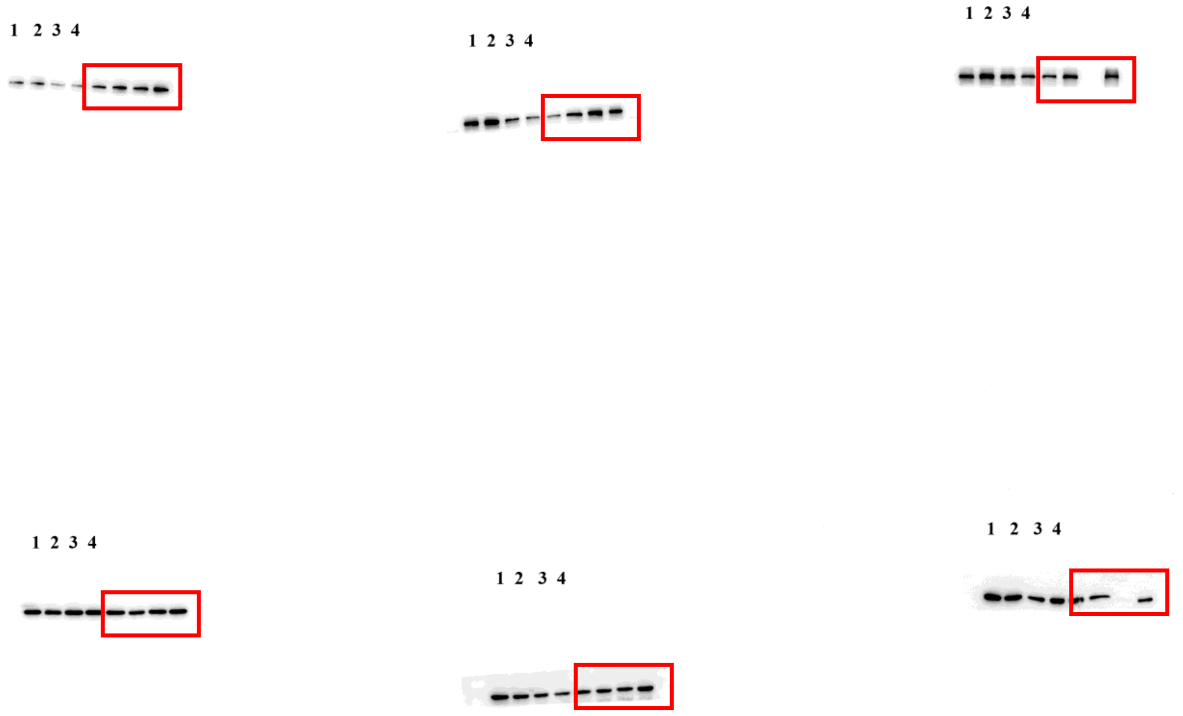

Lane 1: Model
Lane 2: BOL
Lane 3: BOM
Lane 4: BOH
Red frame： These blots were samples of a related project, which are not being used in this manuscript.

Fig. 6 C p-mTOR, and the reference protein.

p-mTOR

GAPDH

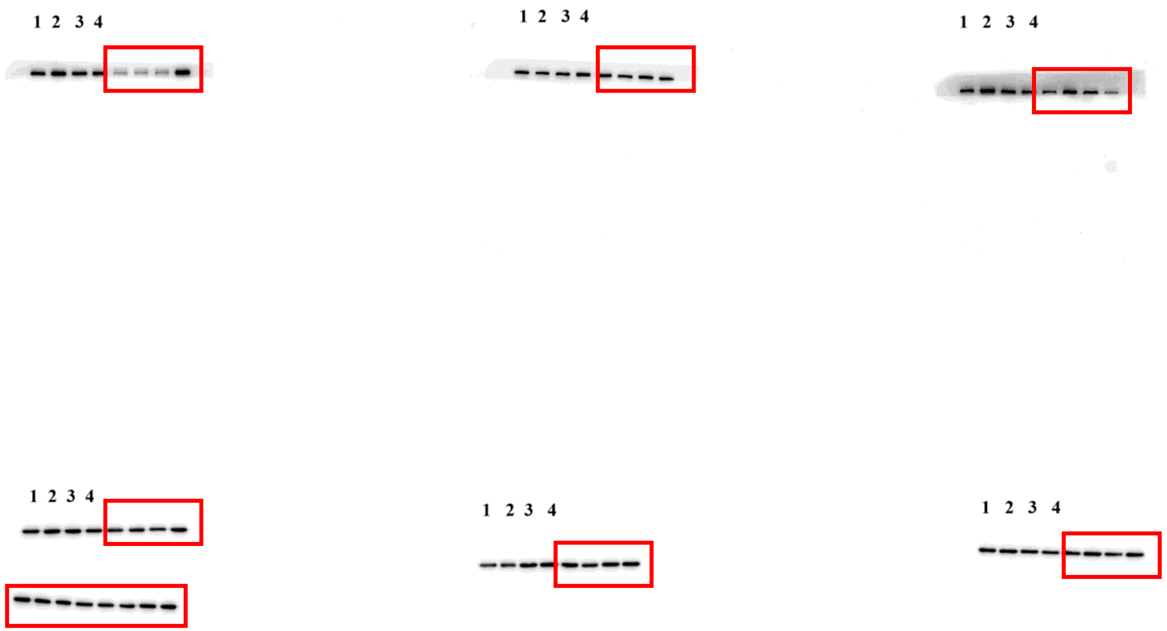

Lane 1: Model
Lane 2: BOL
Lane 3: BOM
Lane 4: BOH
Red frame： These blots were samples of a related project, which are not being used in this manuscript.

Fig. 6 C p-mTOR, and the reference protein.

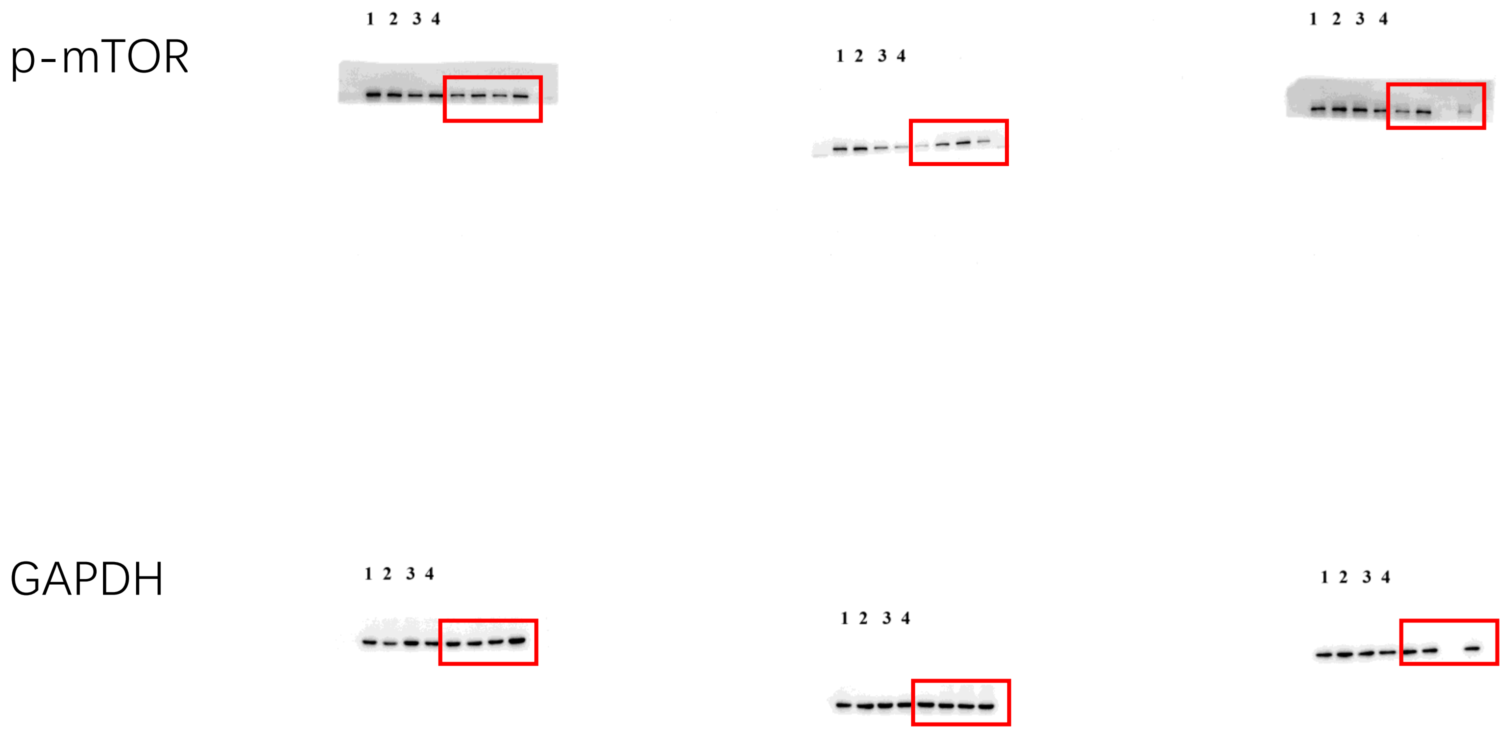

Lane 1: Model
Lane 2: BOL
Lane 3: BOM
Lane 4: BOH
Red frame： These blots were samples of a related project, which are not being used in this manuscript.

Fig. 6 C PI3K, and the reference protein.

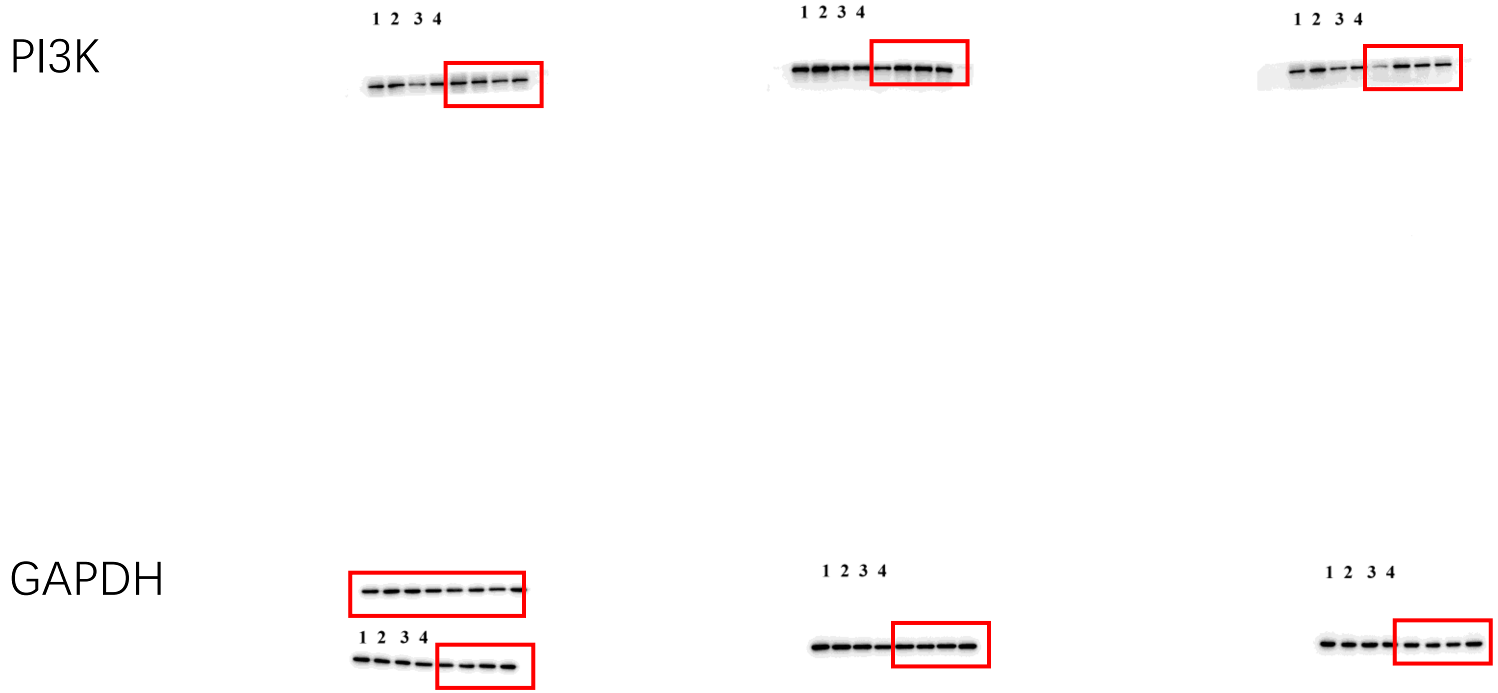

Lane 1: Model
Lane 2: BOL
Lane 3: BOM
Lane 4: BOH
Red frame： These blots were samples of a related project, which are not being used in this manuscript.

Fig. 6 C PI3K, and the reference protein.

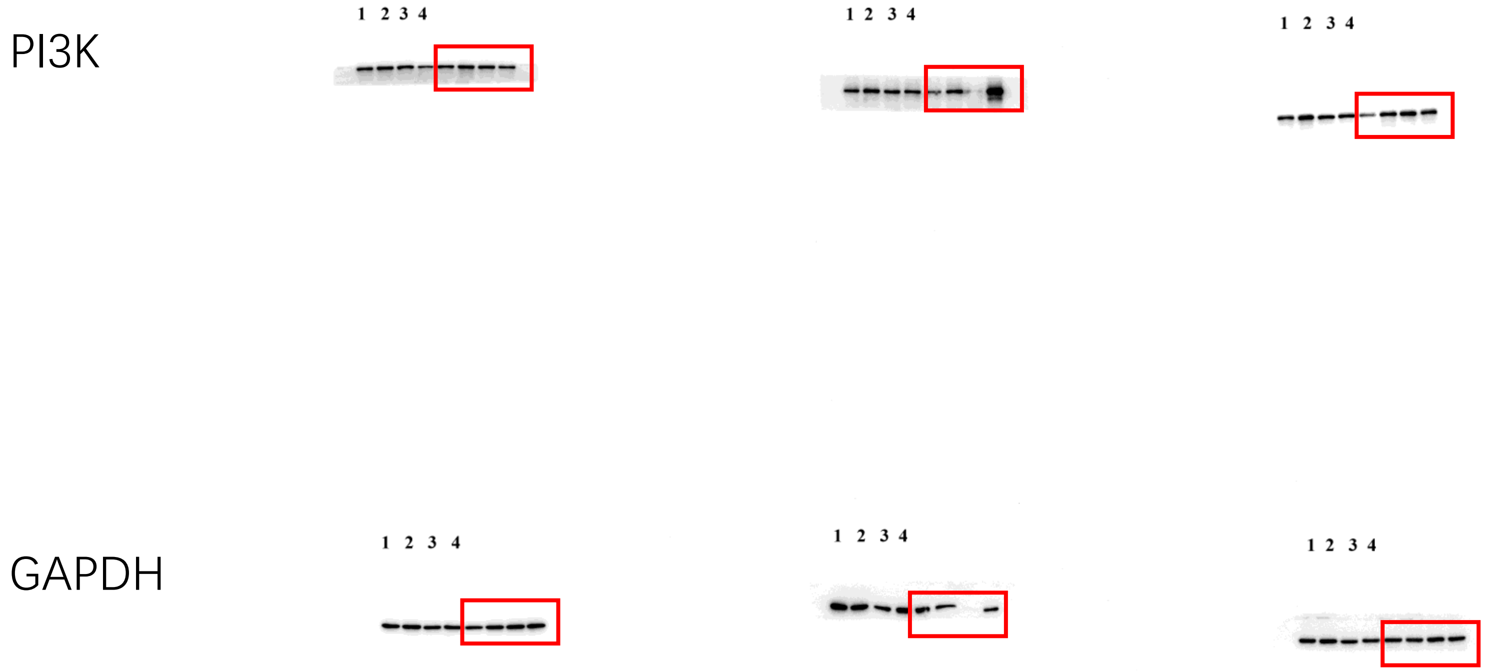

Lane 1: Model
Lane 2: BOL
Lane 3: BOM
Lane 4: BOH
Red frame： These blots were samples of a related project, which are not being used in this manuscript.

Fig. 6 C p-PI3K, and the reference protein.

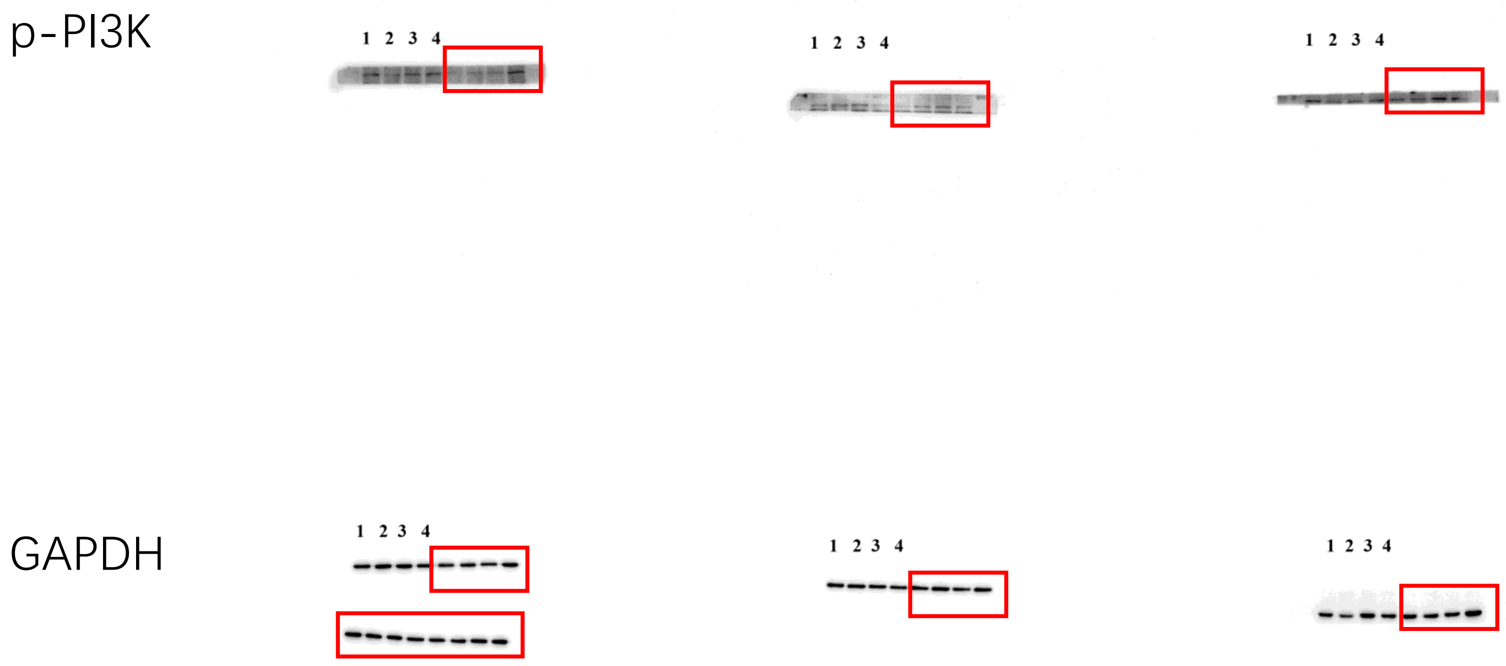

Lane 1: Model
Lane 2: BOL
Lane 3: BOM
Lane 4: BOH
Red frame： These blots were samples of a related project, which are not being used in this manuscript.

Fig. 6 C p-PI3K, and the reference protein.

p-PI3K

GAPDH

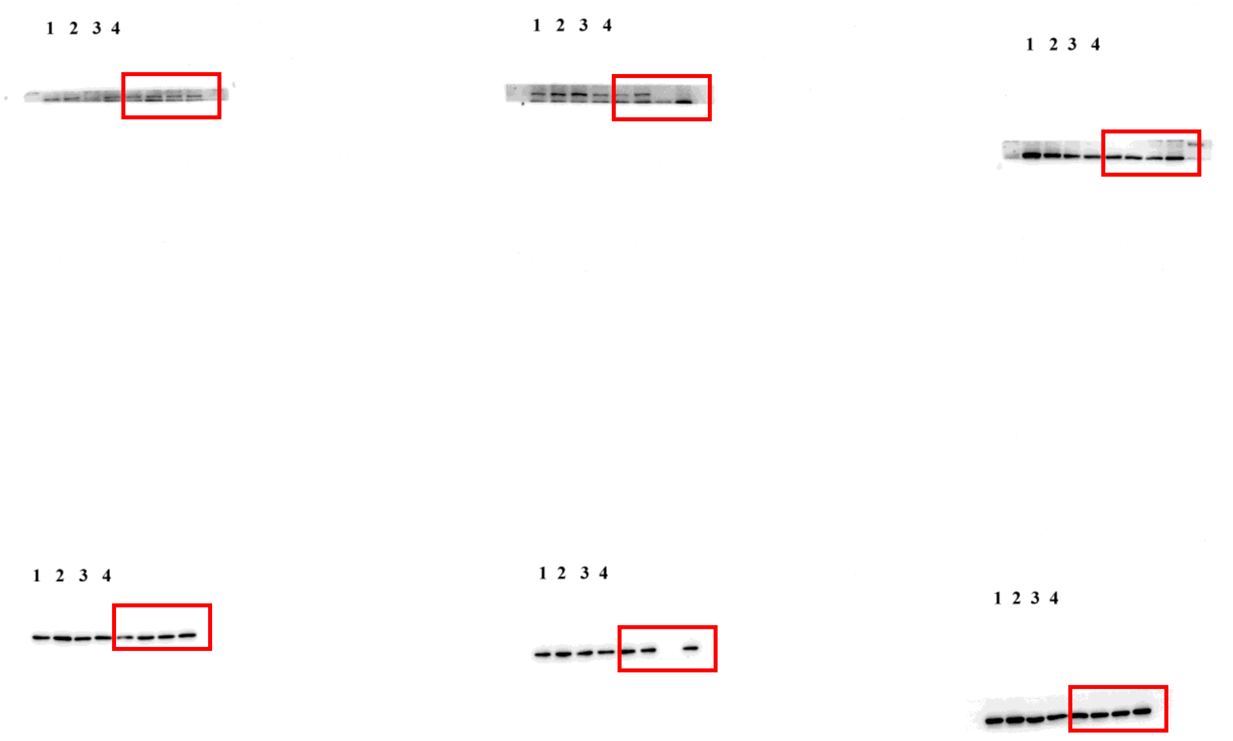

Lane 1: Model
Lane 2: BOL
Lane 3: BOM
Lane 4: BOH
Red frame：These blots were samples of a related project, which are not being used in this manuscript.
