# Supplementary material for: Bruceae Fructus Oil Inhibits Triple-Negative Breast Cancer by Restraining Autophagy: Dependence on the Gut Microbiota-Mediated Amino Acid Regulation
Source: Front Pharmacol. 2021 Oct 1;12:727082. doi: 10.3389/fphar.2021.727082 (PMC8517338; doi:10.3389/fphar.2021.727082)
Supplement: Supplementary file 1 [file DataSheet1.zip › Supplementary materials and raw data/Raw data/Un-cropped WB images for Fig 7.pdf]

Fig 7 A Beclin-1 and the reference protein (β-Actin)

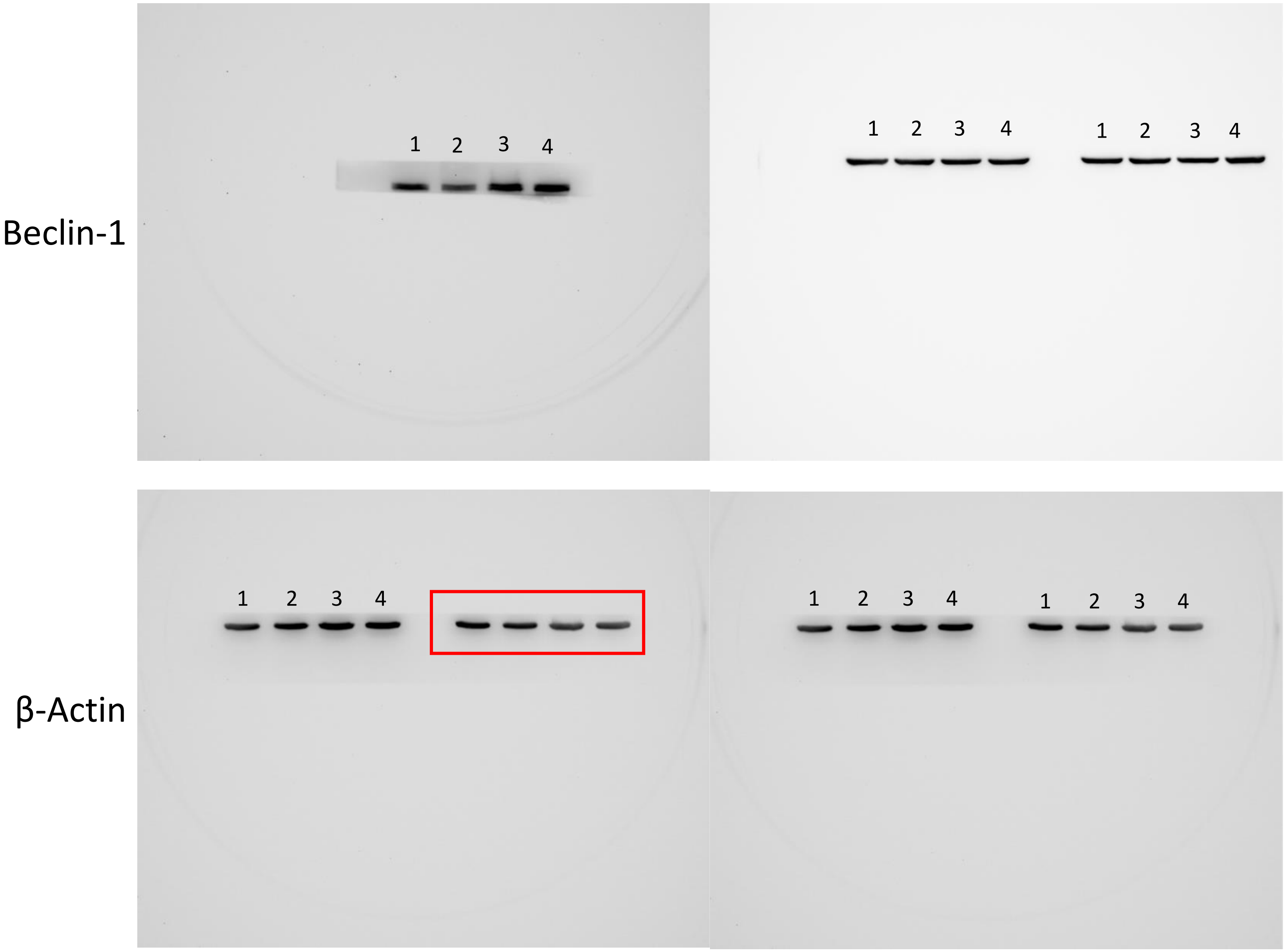

Lane 1: Model
Lane 2: BOH
Lane 3: ABX-Model
Lane 4: ABX-BOH
Red frame： These blots were samples of a related project, which are not being used in this manuscript.

Fig 7 A Beclin-1 and the reference protein (β-Actin)

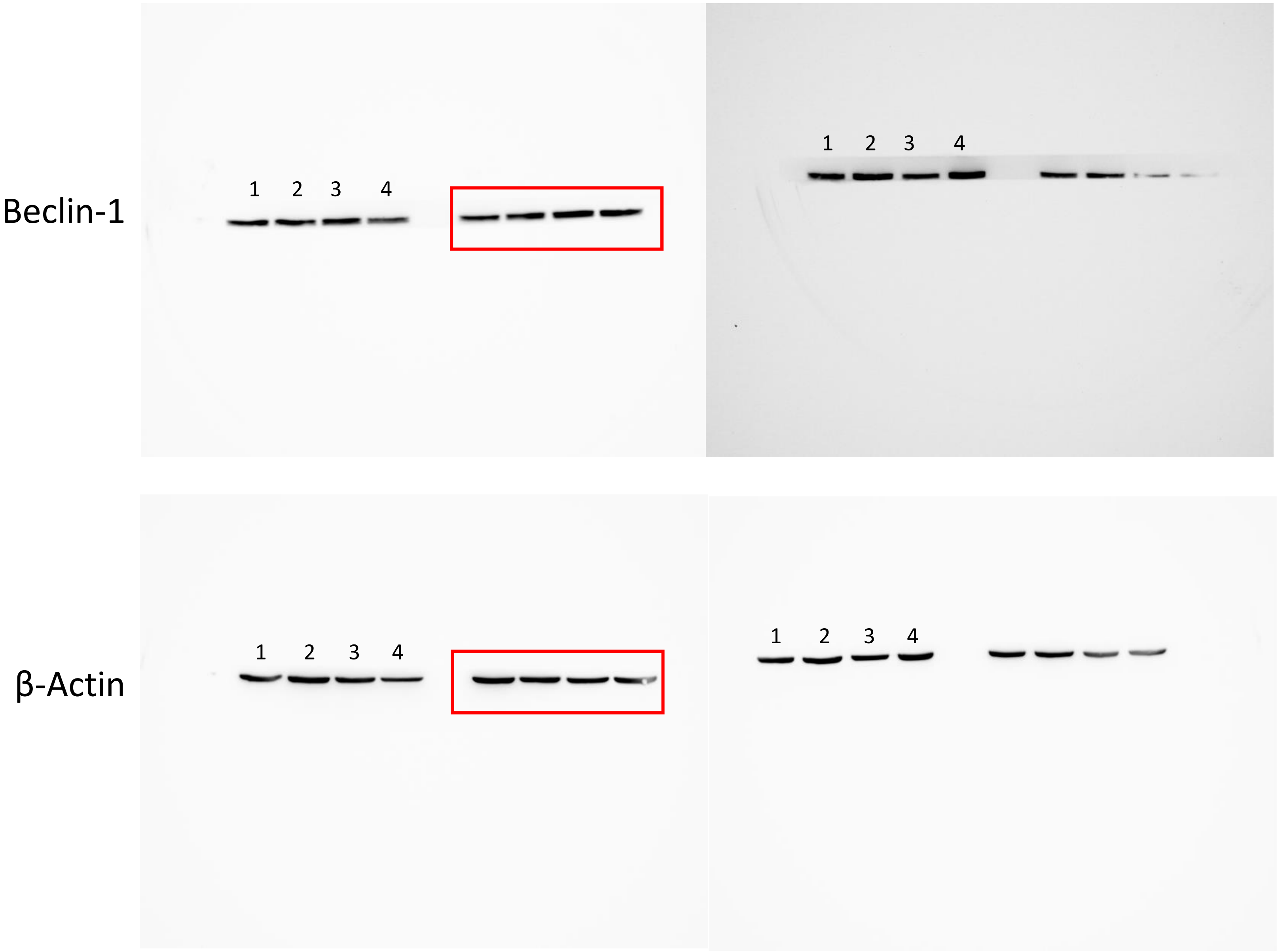

Lane 1: Model
Lane 2: BOH
Lane 3: ABX-Model
Lane 4: ABX-BOH
Red frame： These blots were samples of a related project, which are not being used in this manuscript.

Fig 7 A p62 and the reference protein (β-Actin)

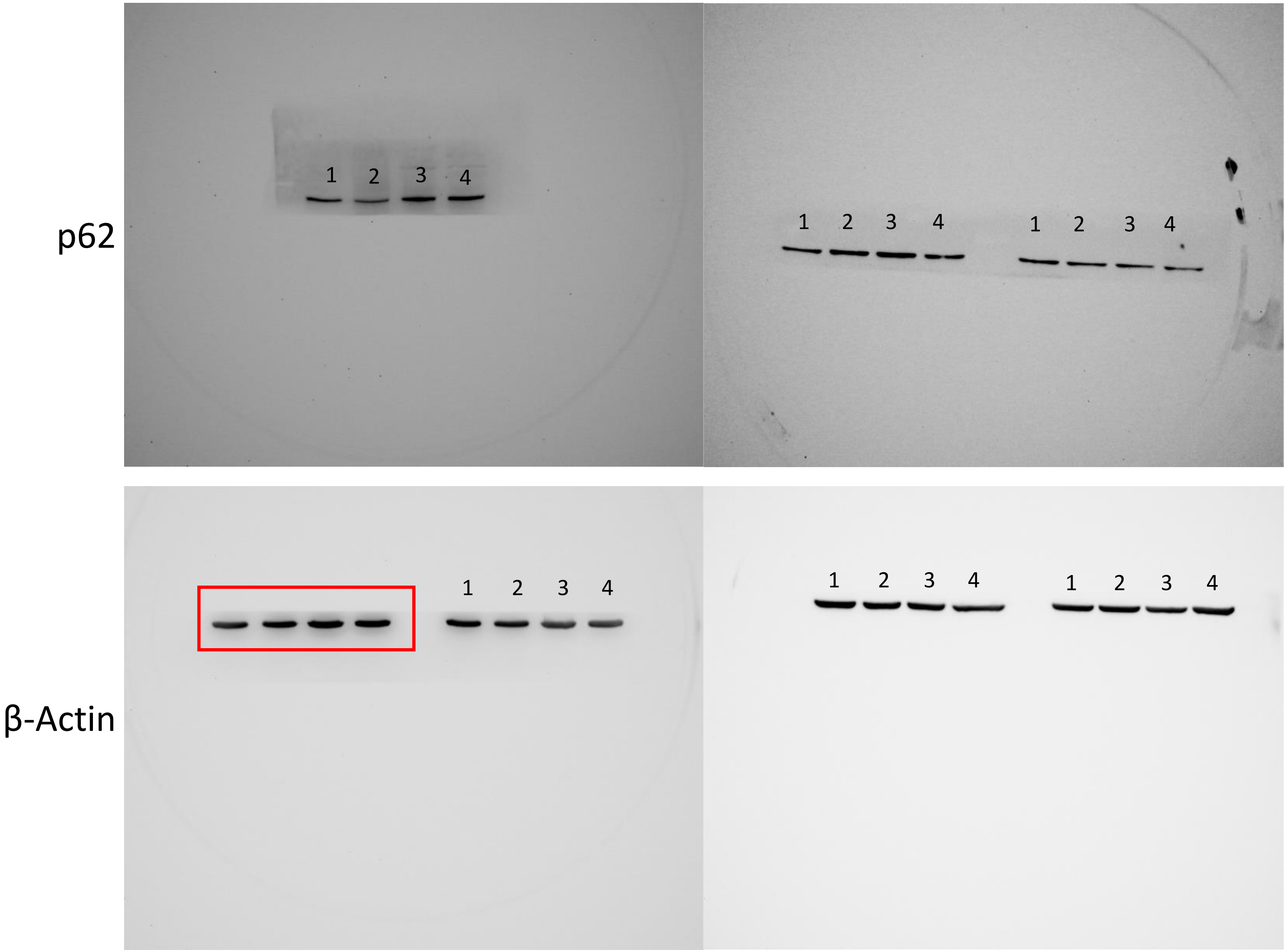

Lane 1: Model
Lane 2: BOH
Lane 3: ABX-Model
Lane 4: ABX-BOH
Red frame： These blots were samples of a related project, which are not being used in this manuscript.

Fig 7 A p62 and the reference protein (β-Actin)

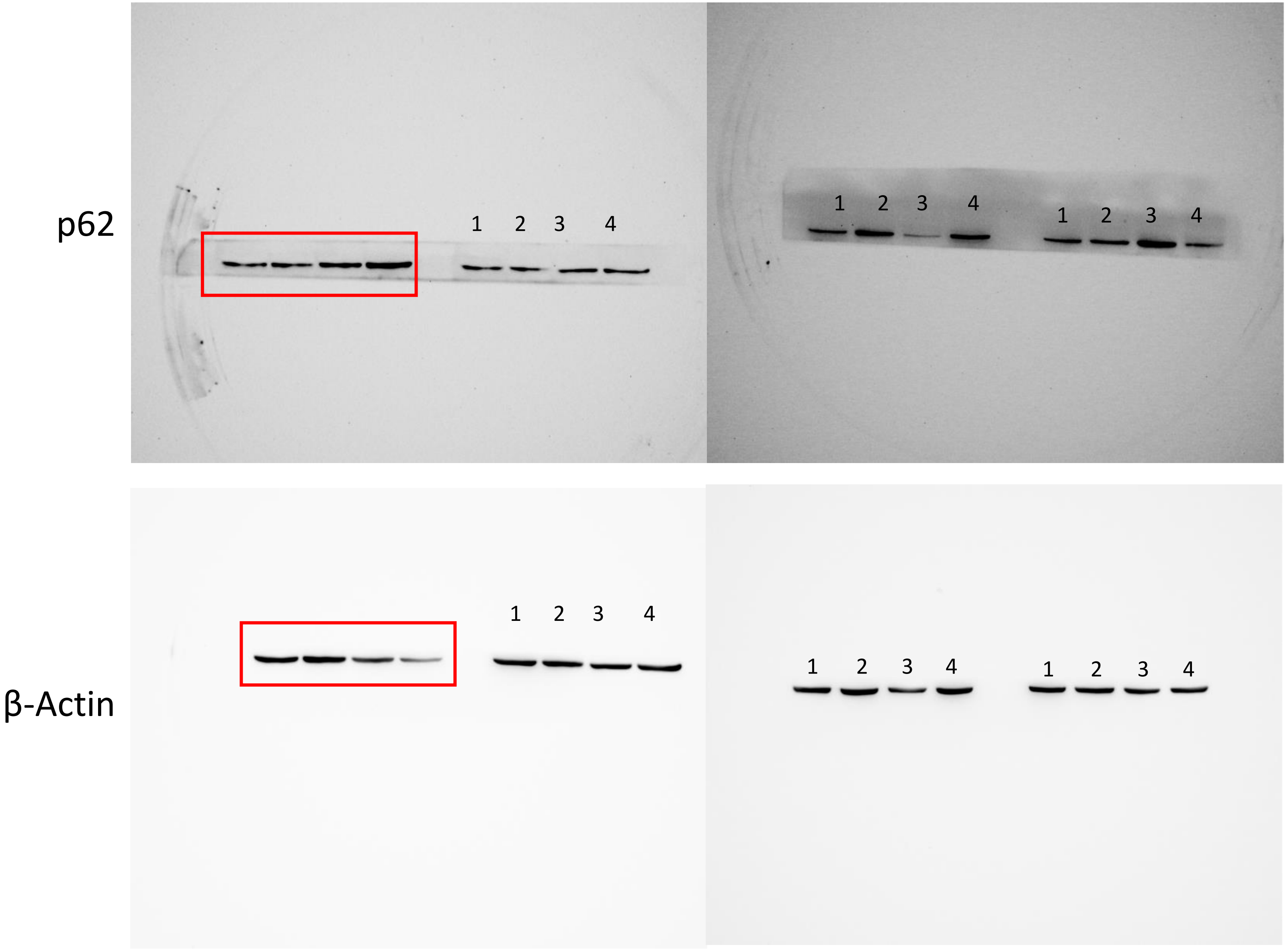

Lane 1: Model
Lane 2: BOH
Lane 3: ABX-Model
Lane 4: ABX-BOH
Red frame： These blots were samples of a related project, which are not being used in this manuscript.

Fig 7 A LC3 II/I and the reference protein (β-Actin)

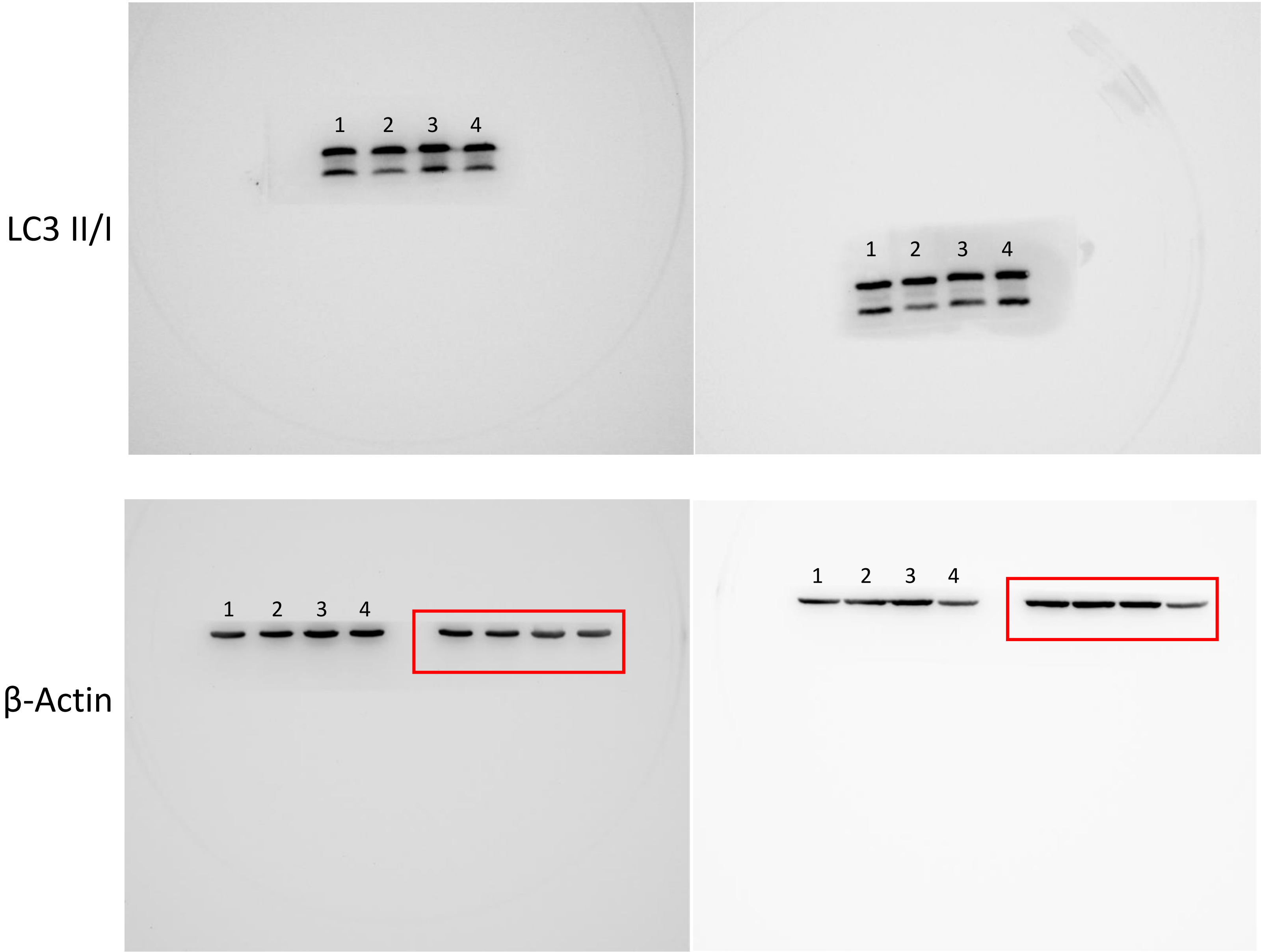

Lane 1: Model

Lane 2: BOH

Lane 3: ABX-Model

Lane 4: ABX-BOH

Red frame： These blots were samples of a related project, which are not being used in this manuscript.

Fig 7 A LC3 II/I and the reference protein (β-Actin)

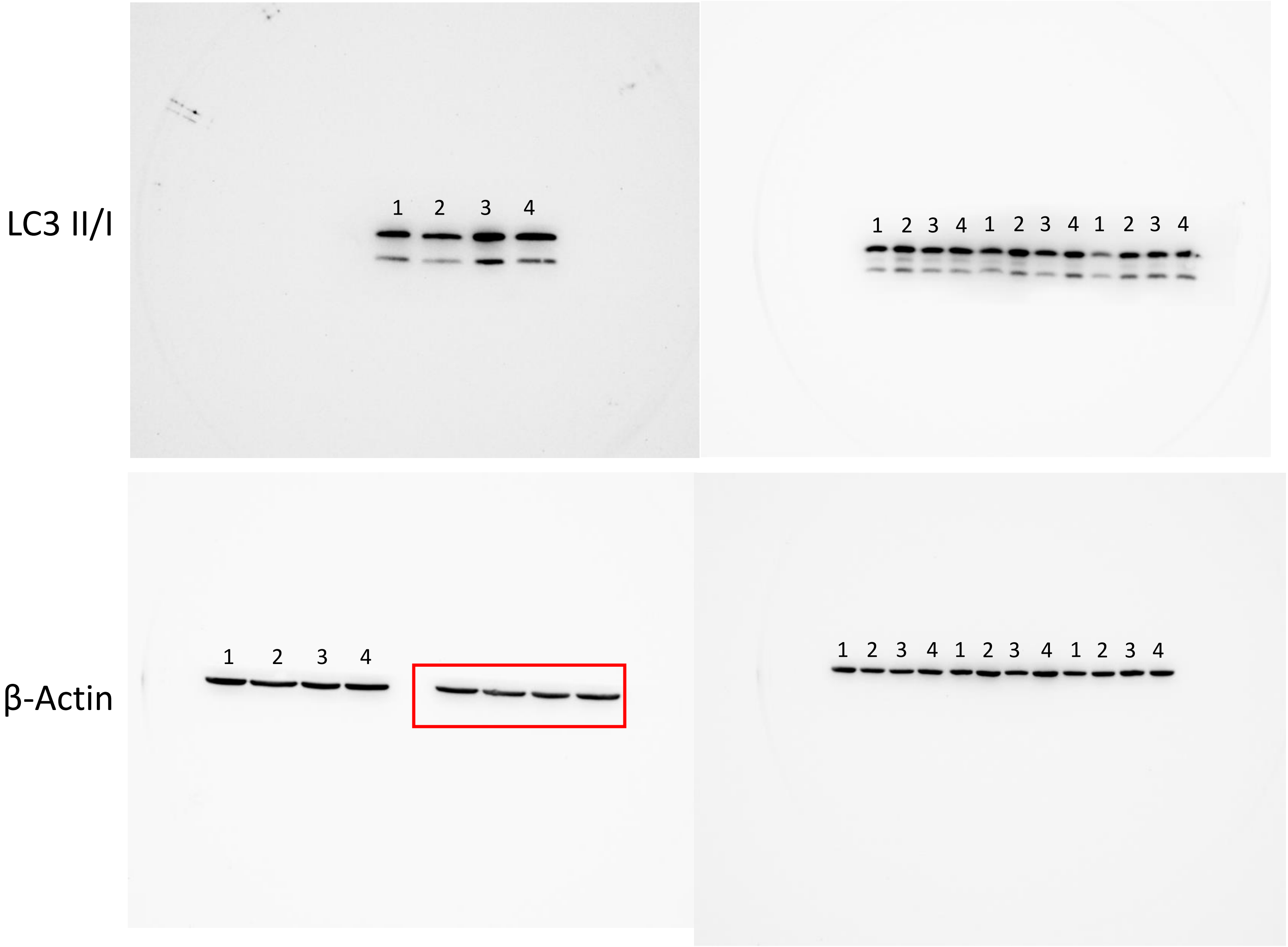

Lane 1: Model
Lane 2: BOH
Lane 3: ABX-Model
Lane 4: ABX-BOH
Red frame： These blots were samples of a related project, which are not being used in this manuscript.

Fig 7 B S6 and the reference protein (β-Actin)

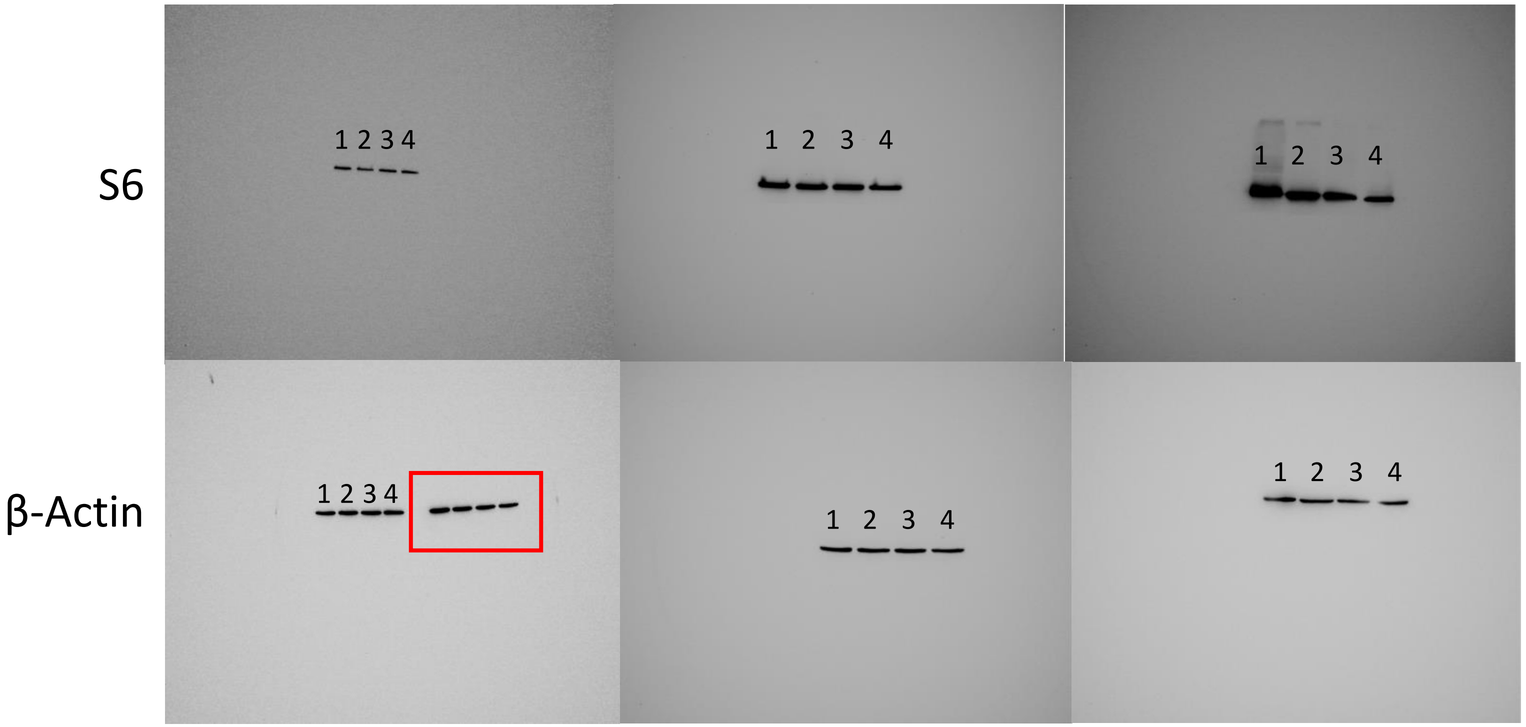

Lane 1: Model
Lane 2: BOH
Lane 3: ABX-Model
Lane 4: ABX-BOH
Red frame： These blots were samples of a related project, which are not being used in this manuscript.

Fig 7 B S6 and the reference protein (β-Actin)

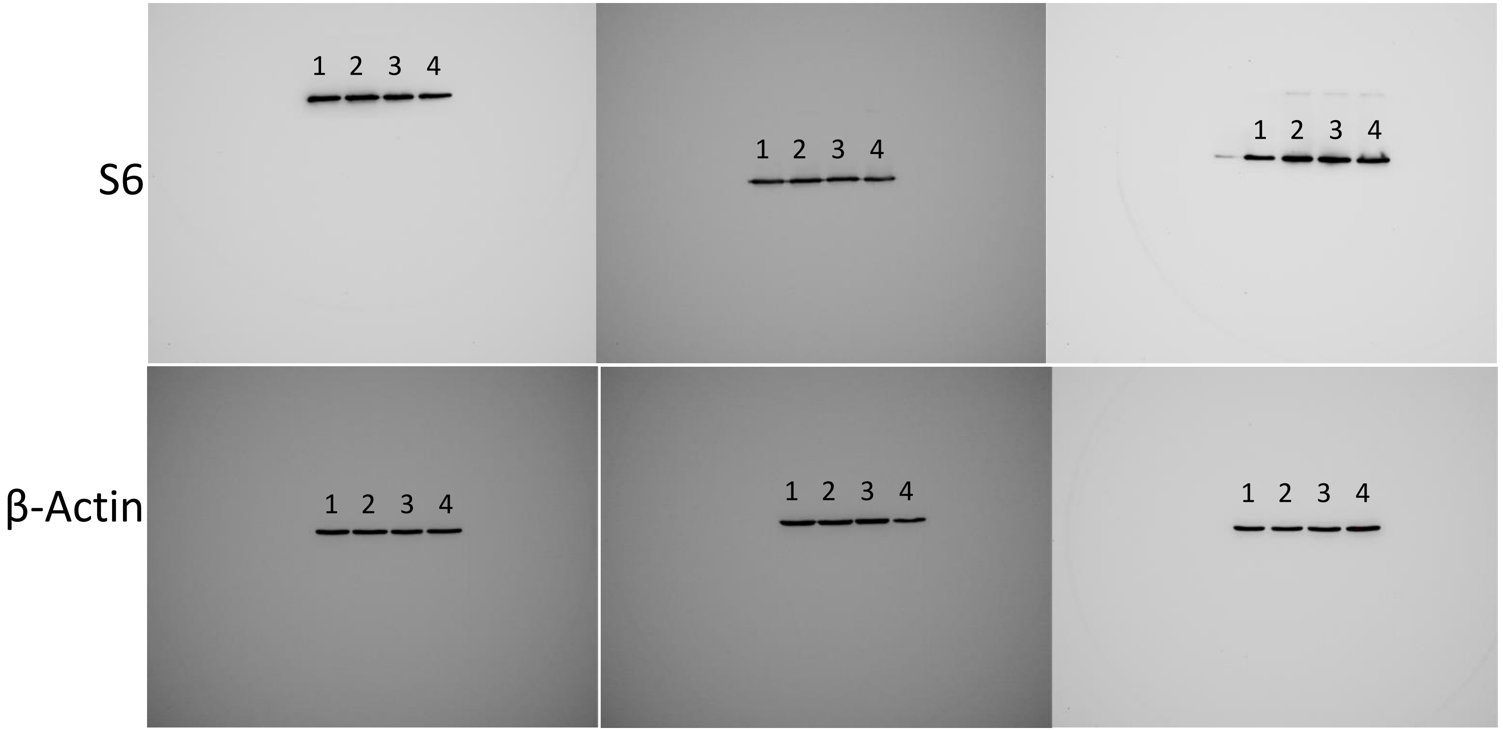

Lane 1: Model
Lane 2: BOH
Lane 3: ABX-Model
Lane 4: ABX-BOH

Fig 7 B p-S6 and the reference protein (β-Actin)

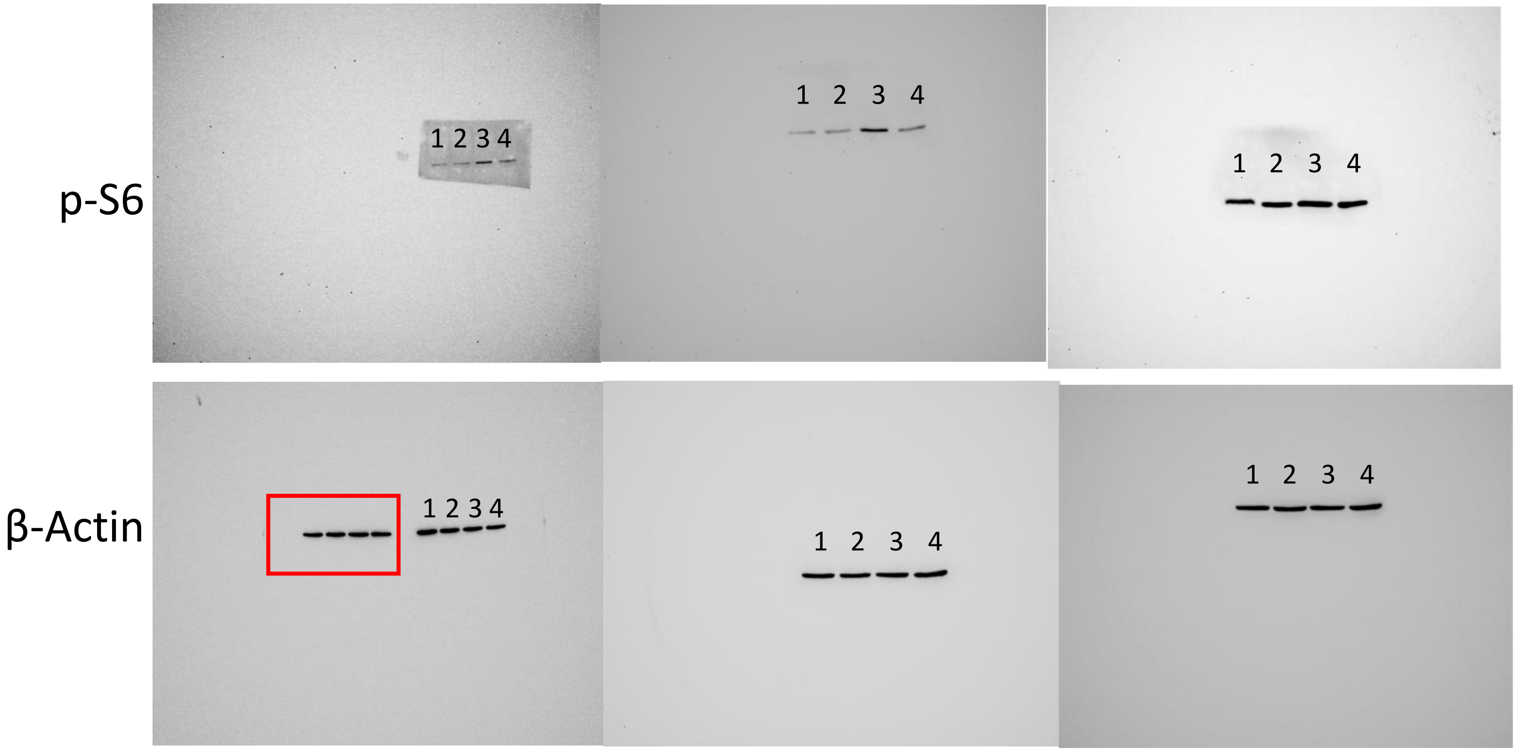

Lane 1: Model
Lane 2: BOH
Lane 3: ABX-Model
Lane 4: ABX-BOH
Red frame： These blots were samples of a related project, which are not being used in this manuscript.

Fig 7 B p-S6 and the reference protein (β-Actin)

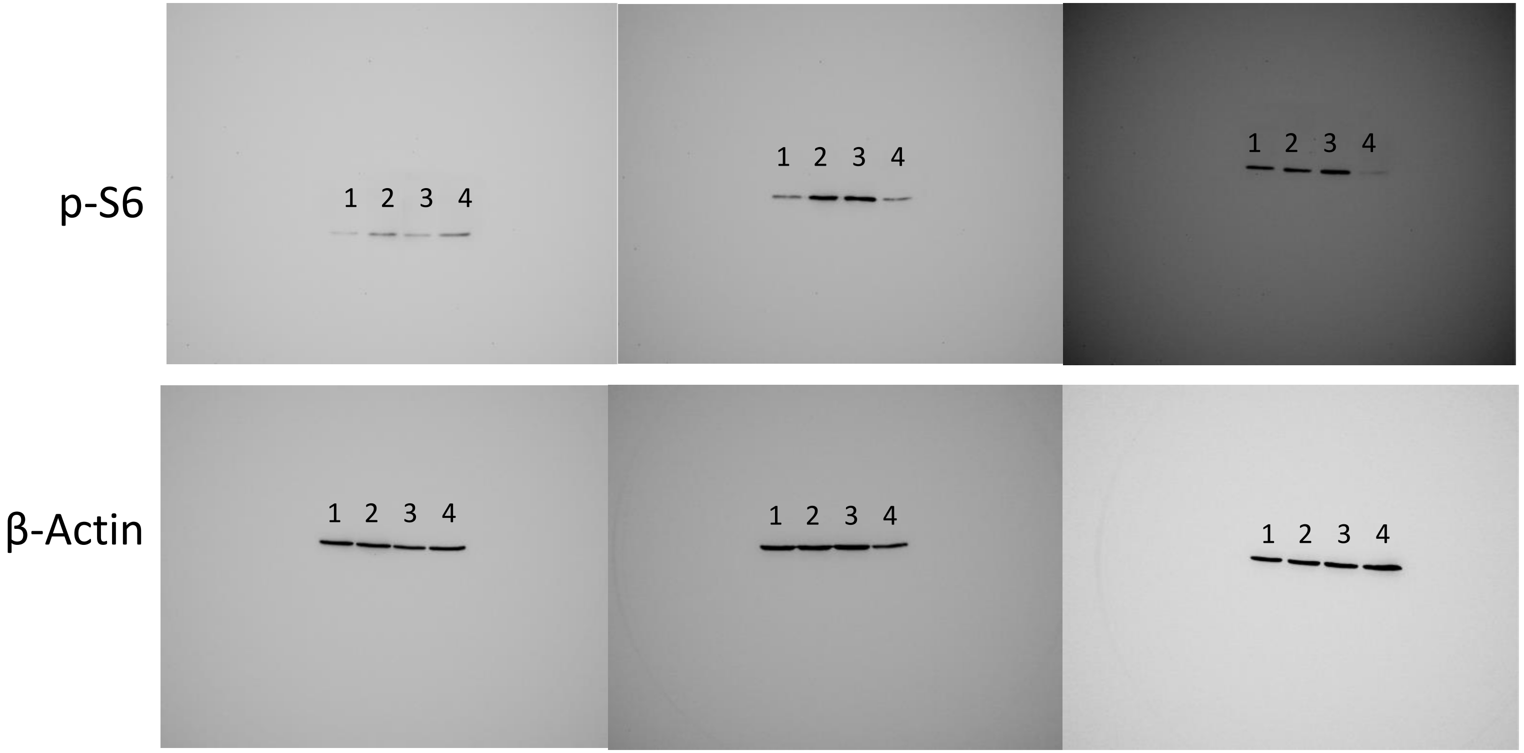

Lane 1: Model
Lane 2: BOH
Lane 3: ABX-Model
Lane 4: ABX-BOH

Fig 7 B 4E-BP1 and the reference protein (β-Actin)

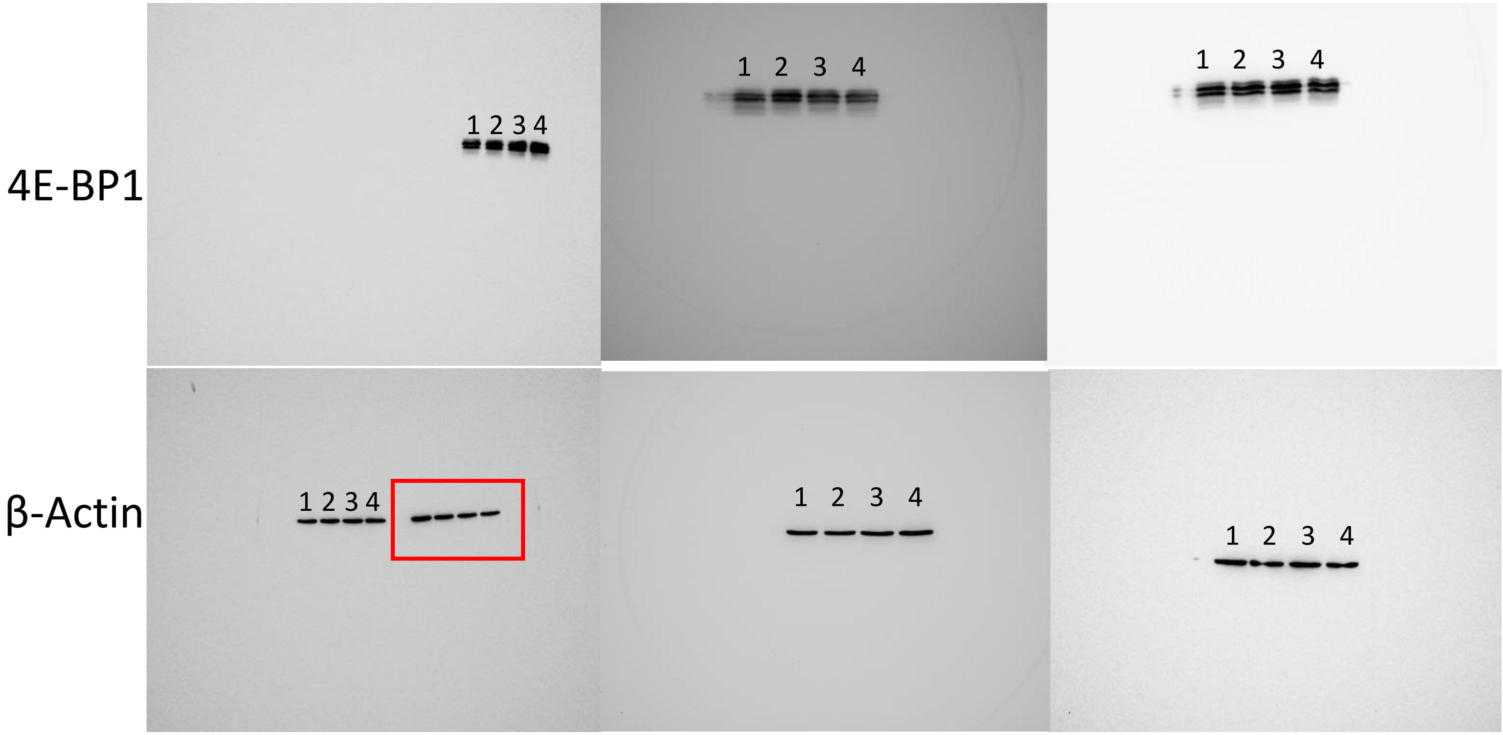

Lane 1: Model
Lane 2: BOH
Lane 3: ABX-Model
Lane 4: ABX-BOH
Red frame： These blots were samples of a related project, which are not being used in this manuscript.

Fig 7 B 4E-BP1 and the reference protein (β-Actin)

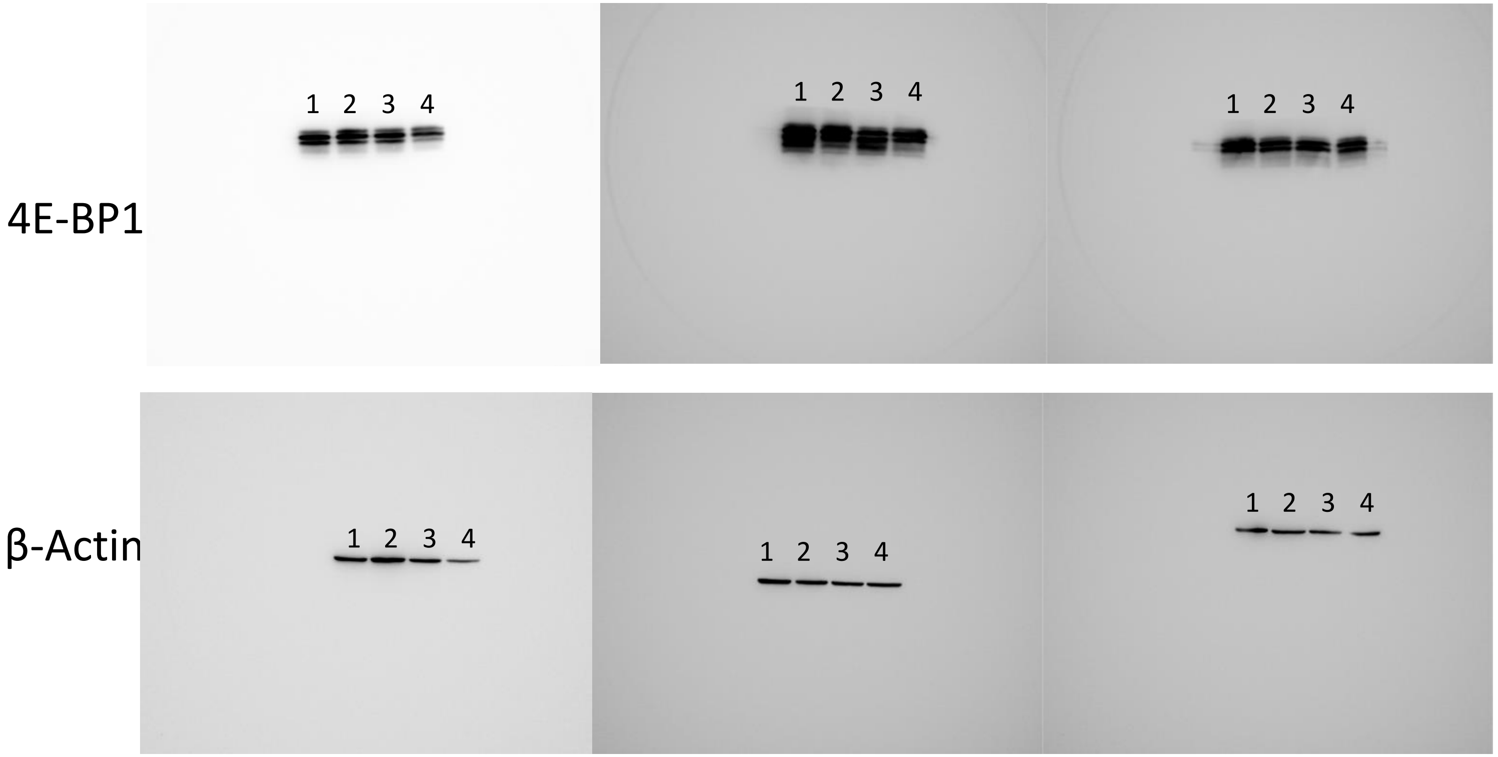

Lane 1: Model
Lane 2: BOH
Lane 3: ABX-Model
Lane 4: ABX-BOH

Fig 7 B p-4E-BP1 and the reference protein (β-Actin)

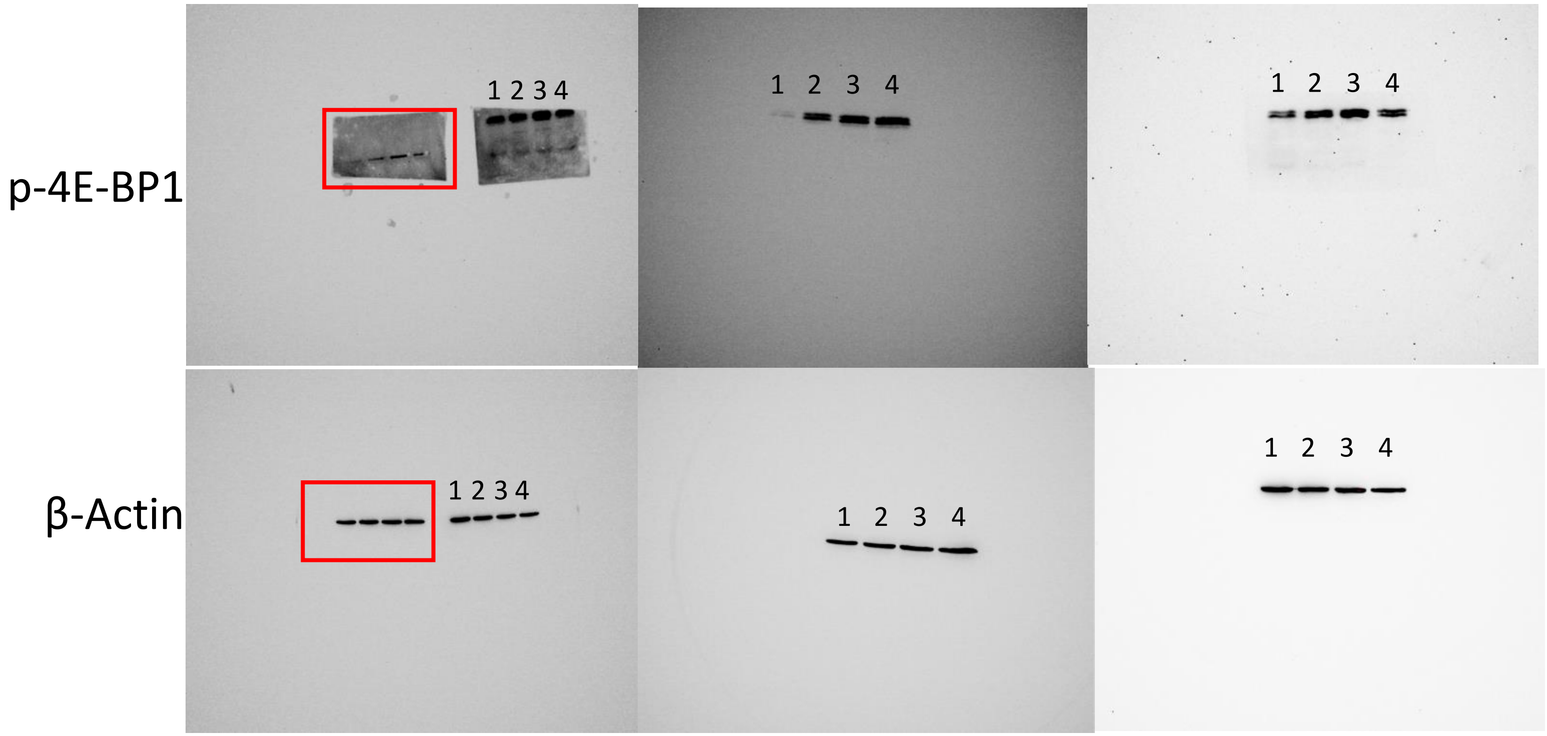

Lane 1: Model
Lane 2: BOH
Lane 3: ABX-Model
Lane 4: ABX-BOH
Red frame： These blots were samples of a related project, which are not being used in this manuscript.

Fig 7 B p-4E-BP1 and the reference protein (β-Actin)

p-4E-BP1

β-Actin

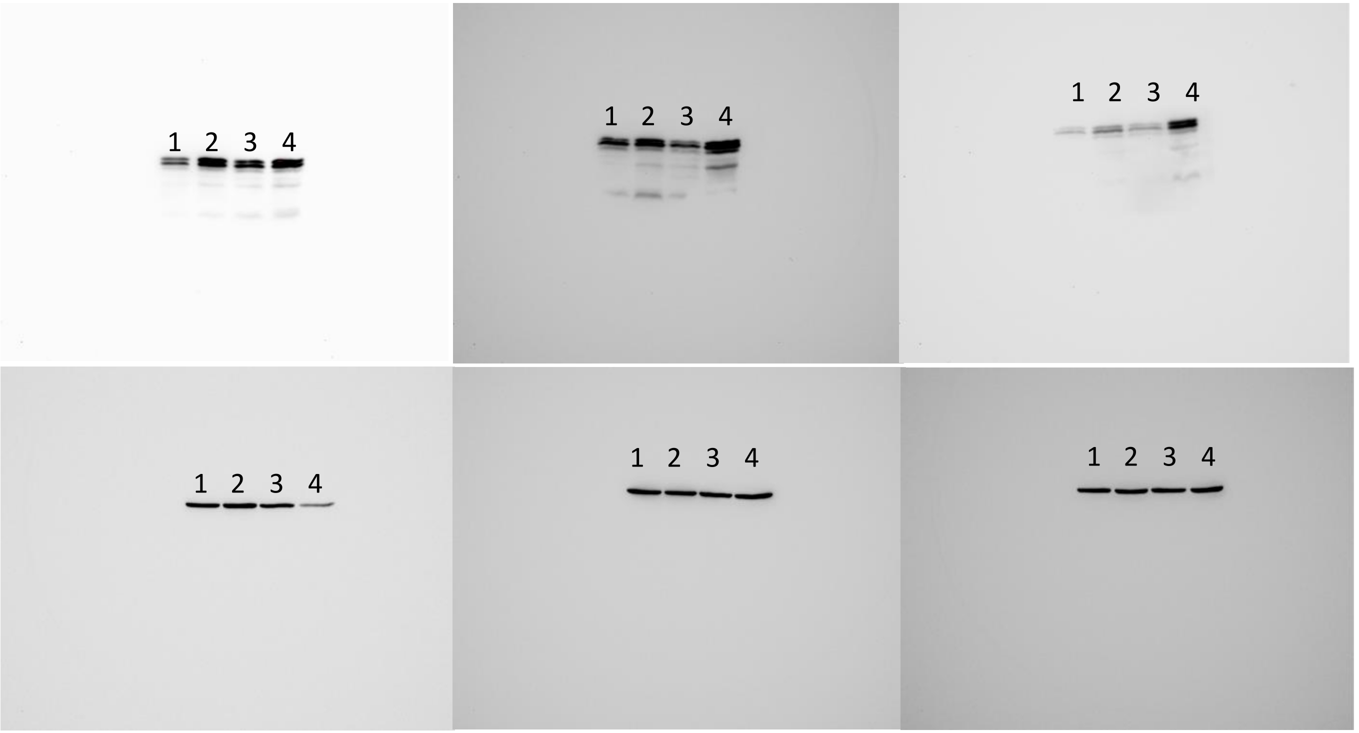

Lane 1: Model
Lane 2: BOH
Lane 3: ABX-Model
Lane 4: ABX-BOH
