# Supplementary material for: Bruceae Fructus Oil Inhibits Triple-Negative Breast Cancer by Restraining Autophagy: Dependence on the Gut Microbiota-Mediated Amino Acid Regulation
Source: Front Pharmacol. 2021 Oct 1;12:727082. doi: 10.3389/fphar.2021.727082 (PMC8517338; doi:10.3389/fphar.2021.727082)
Supplement: Supplementary file 1 [file DataSheet1.zip › Supplementary materials and raw data/Supporting information.docx]

**Bruceae fructus oil inhibits triple-negative breast cancer** **by restraining autophagy: Dependence on the gut microbiota-mediated amino acid regulation**

Jiyan Su ^1^ †, Xiaohong Chen ^2, 3^ †, Yuanjie Xiao ^4^, Dan Li ^5^, Muxia Li ^5^, Hongfu Li ^6^, Jiangjian Huang ^7^, Zhengquan Lai ^8^, Ziren Su ^5^, Yizhen Xie ^3^, Dajiang Zhu ^1^, Qianjun Chen ^9^, Hai Lu ^9^, Jingjin He ^6, 10^ *, Chenglai Xia ^1, 10^ *

1. Affiliated Foshan Maternity & Child Healthcare Hospital, Southern Medical University, Foshan 528000, Guangdong, P.R. China
2. Department of Basic Medical Science, Xiamen Medical College, Xiamen, 361023, Fujian, P. R. China
3. Guangdong Provincial Key Laboratory of Microbial Safety and Health, State Key Laboratory of Applied Microbiology Southern China, Guangdong Institute of Microbiology, Guangdong Academy of Science, Guangzhou, 510070, Guangdong, P.R. China
4. Department of Cell Biology & Institute of Biomedicine, College of Life Science and Technology, Jinan University, Guangzhou, 510632, Guangdong, P.R. China
5. School of Pharmaceutical Science, Guangzhou University of Chinese Medicine, Guangzhou, 510006, Guangdong, P.R. China
6. The Eighth Affiliated Hospital, Sun Yat-sen University, Shenzhen 518033, Guangdong, P. R. China
7. Guangzhou Baiyunshan Ming xing Pharmaceutical Co., Ltd.，Guangzhou 510250, Guangdong, P. R. China
8. Department of Pharmacy, Shenzhen University General Hospital, Shenzhen University, Shenzhen, 518061, Guangdong, P. R. China.
9. Department of Breast Disease, Guangdong Provincial Hospital of Chinese Medicine, Guangzhou, 510006, Guangdong, P.R. China.
10. Shenzhen International Institute for Biomedical Research, Shenzhen, China
11. School of Pharmaceutical Sciences, Southern Medical University, Guangzhou 510150, Guangdong, P. R. China

† These authors contributed equally to this work

^*^ **Correspondence**

Jingjin He, Research Assistant, The Eighth Affiliated Hospital, Sun Yat-sen University. E-mail: he_jingjin@hotmail.com.

Chenglai Xia, Professor, Affiliated Foshan Maternity & Child Healthcare Hospital, School of Pharmaceutical Sciences, Southern Medical University. E-mail: xiachenglai@126.com.

**Supplement methods**

1. Fatty acids determination for BO by gas chromatography-mass spectrometry (GC-MS)
   1. Standard mixture preparation

Standard mixture stocking solution of the 46 fatty acid methyl esters was diluted with n-hexane as the following series concentrations (total concentration for all the standards): 1, 5, 10, 25, 50, 100, 250, 500, 1000, 2000 μg/mL, and detail information of each fatty acid methyl esters and the corresponding proportion were listed in the following Table S3.

- 1. Sample preparation

One hundred μL BO was mixed with 2mL 1% methanol (in sulfuric acid), votexed for 1 min, and subjected to esterification for 30 min at 80℃. Then it was extracted with 1mL n-hexane, washed with 5mL distilled water at 4℃, and centrifuged (12000 rpm, 4℃, 10 min). 700 μL of the supernatant was dehydrated with 100mg anhydrous sodium sulfate by votex (30s) and centrifuged (12000 rpm, 4℃, 10 min). 100 μL of the obtained supernatant was diluted with 900 μL n-hexane, and then mixed with 15 μL 500 ppm methyl salicylate (as internal standard) for detection.

- 1. GC-MS detection

The samples were analyzed with Trace 1310-ISQ 7000 GC-MS (Thermo Fisher Scientific, Waltham, MA, USA). Chromatography analysis was performed on Thermo TG-FAME capillary column (50 m*0.25 mm ID*0.20 μm). Injection volume was 1 μL, and split ratio was 8 : 1. Temperature of injection port, ion source, transmission line, and quadrupole rod was 250℃, 230℃, 250℃, and 150℃, respectively. The temperature program was as follows: 80℃ for 1 min, up to 160℃ for 1.5 min by 20℃/min, up to 196℃ for 8.5 min by 3℃/min, and then up to 250℃ for 3 min by 20℃/min. The carrier gas was helium (He) with 0.63 mL/min. Mass spectrum was performed with electron impact ionization source by single ion monitoring mode, and the collision energy was 70 eV. Quantification parameters were showed in Table S4.

**Supplement Tables**

Table S1 Proportion of each fatty acid in the standard mixture

| Abbreviation | Name | Proportion（%） |
| --- | --- | --- |
| C6:0 | Caproate | 2.0 |
| C8:0 | Caprylate | 2.0 |
| C10:0 | Caprate | 1.0 |
| C12:0 | Laurate | 1.0 |
| C14:0 | Myristate | 2.0 |
| C14:1T | Myristelaidate | 1.0 |
| C14:1 | Myristoleate | 2.0 |
| C15:0 | Pentadecanoate | 2.0 |
| C15:1T | 10-Transpentadecenoate | 1.0 |
| C15:1 | 10-Pentadecenoate | 2.0 |
| C16:0 | Palmitate | 3.0 |
| C16:1T | Palmitelaidate | 1.0 |
| C16:1 | Palmitoleate | 2.0 |
| C17:0 | Heptadecanoate | 3.0 |
| C17:1T | 10-Transsheptadecenoate | 2.0 |
| C17:1 | 10-Heptadecenoate | 2.0 |
| C18:0 | Stearate | 2.0 |
| C18:1N9T | Elaidate | 3.0 |
| C18:1N7T | Transvaccenate | 1.0 |
| C18:1N9C | Oleate | 2.0 |
| C18:1N7 | Vaccenate | 2.0 |
| C18:2N6T | Linoelaidate | 2.0 |
| C19:1N12T | 7-Transnonadecenoate | 1.0 |
| C18:2N6 | Linoleate | 3.0 |
| C20:0 | Arachidate | 2.0 |
| C18:3N6 | Gamma Linolenate | 3.0 |
| C20:1T | Trans 11-Eicosenoate | 1.0 |
| C20:1 | 11-Eicosenoate | 2.0 |
| C18:3N3 | Alpha Linolenate | 3.0 |
| C21:0 | Heneicosanoate | 2.0 |
| C20:2 | 11-14 Eicosadienoate | 3.0 |
| C22:0 | Behenate | 1.0 |
| C20:3N6 | Homogamma Linolenate | 2.0 |
| C22:1N9T | Brassidate | 1.0 |
| C22:1N9 | Erucate | 2.0 |
| C20:3N3 | 11-14-17 Eicosatrienoate | 2.0 |
| C20:4N6 | Arachidonate | 3.0 |
| C23:0 | Tricosanoate | 2.0 |
| C22:2 | Docosadienoate | 2.0 |
| C20:5N3 | Eicosapentaenoate | 2.0 |
| C24:0 | Lignocerate | 1.0 |
| C24:1 | Nervonoate | 1.0 |
| C22:4 | Docosatetraenoate | 2.0 |
| C22:5N6 | Docosapentaenoate | 2.0 |
| C22:5N3 | Docosapentaenoate | 2.0 |
| C22:6N3 | Docosahexaenoate | 2.0 |

Table S2 Quantification parameters

| Name | Retention  （min） | Quantitative ion  （m/z） | Equation of linear regression | Correlation coefficient（*r*） | Linear range（μg/mL） |
| --- | --- | --- | --- | --- | --- |
| C6:0 | Caproate | 5.1 | 74 | y=0.0501x + 0.00053 | 0.993 |
| C8:0 | Caprylate | 6.13 | 74 | y=0.0959x + 0.00051 | 0.994 |
| C10:0 | Caprate | 7.33 | 74 | y=0.129x + 3e-04 | 0.992 |
| C12:0 | Laurate | 8.76 | 74 | y=0.126x + 0.00037 | 0.992 |
| C14:0 | Myristate | 10.58 | 120 | y=0.105x + 0.00038 | 0.99 |
| C14:1 | Myristoleate | 11.46 | 69 | y=0.0427x + 0.00402 | 0.991 |
| C14:1T | Myristelaidate | 11.16 | 74 | y=0.0467x + 0.00471 | 0.992 |
| C15:0 | Pentadecanoate | 11.69 | 69 | y=0.101x + 0.00029 | 0.99 |
| C15:1 | 10-Pentadecenoate | 12.68 | 69 | y=0.0426x + 0.00223 | 0.992 |
| C15:1T | 10-Transpentadecenoate | 12.35 | 74 | y=0.0477x + 0.00163 | 0.998 |
| C16:0 | Palmitate | 12.94 | 69 | y=0.0942x + 0.00167 | 0.992 |
| C16:1 | Palmitoleate | 13.84 | 69 | y=0.0333x + 0.00295 | 0.996 |
| C16:1T | Palmitelaidate | 13.55 | 74 | y=0.0349x + 0.00234 | 0.992 |
| C17:0 | Heptadecanoate | 14.33 | 69 | y=0.0872x + 4e-04 | 0.995 |
| C17:1 | 10-Heptadecenoate | 15.29 | 69 | y=0.0341x + 0.00279 | 0.996 |
| C17:1T | 10-Transsheptadecenoate | 14.99 | 74 | y=0.0336x + 0.00274 | 0.99 |
| C18:0 | Stearate | 15.83 | 69 | y=0.0843x + 0.00238 | 0.996 |
| C18:1N7 | Vaccenate | 16.84 | 69 | y=0.0333x + 0.00219 | 0.991 |
| C18:1N7T | Transvaccenate | 16.52 | 69 | y=0.0114x + 0.00208 | 0.991 |
| C18:1N9C | Oleate | 16.71 | 69 | y=0.0307x + 0.00314 | 0.993 |
| C18:1N9T | Elaidate | 16.45 | 69 | y=0.0912x + 0.00249 | 0.993 |
| C18:2N6 | Linoleate | 18.14 | 74 | y=0.054x + 0.00222 | 0.99 |
| C18:2N6T | Linoelaidate | 17.51 | 69 | y=0.0378x + 0.00179 | 0.996 |
| C18:3N3 | Alpha Linolenate | 20.04 | 69 | y=0.00264x + 0.00083 | 0.996 |
| C18:3N6 | Gamma Linolenate | 19.23 | 74 | y=0.0301x + 0.00114 | 0.996 |
| C19:1N12T | 7-Transnonadecenoate | 17.98 | 67 | y=0.0306x + 0.00243 | 0.99 |
| C20:0 | Arachidate | 19.09 | 67 | y=0.0671x + 0.00071 | 0.995 |
| C20:1 | 11-Eicosenoate | 19.92 | 69 | y=0.0204x + 0.00268 | 0.993 |
| C20:1T | Trans 11-Eicosenoate | 19.73 | 79 | y=0.0284x + 0.0018 | 0.998 |
| C20:2 | 11-14 Eicosadienoate | 21.79 | 74 | y=0.027x + 0.00196 | 0.994 |
| C20:3N3 | 11-14-17 Eicosatrienoate | 24.18 | 69 | y=0.0271x + 0.00114 | 0.996 |
| C20:3N6 | Homogamma Linolenate | 23.22 | 74 | y=0.022x + 0.001 | 0.993 |
| C20:4N6 | Arachidonate | 24.45 | 79 | y=0.0224x + 0.00114 | 0.994 |
| C20:5N3 | Eicosapentaenoate | 27.51 | 67 | y=0.0241x + 0.00108 | 0.992 |
| C21:0 | Heneicosanoate | 20.98 | 79 | y=0.053x + 0.00042 | 0.994 |
| C22:0 | Behenate | 23.22 | 67 | y=0.0427x + 0.00022 | 0.992 |
| C22:1N9 | Erucate | 24.45 | 69 | y=0.0298x + 0.00254 | 0.991 |
| C22:1N9T | Brassidate | 24.07 | 79 | y=0.0206x + 0.0018 | 0.998 |
| C22:2 | Docosadienoate | 26.92 | 74 | y=0.0186x + 0.00178 | 0.995 |
| C22:4 | Docosatetraenoate | 29.12 | 55 | y=0.0423x + 0.00245 | 0.994 |
| C22:5N3 | Docosapentaenoate | 30.36 | 79 | y=0.0545x + 0.00437 | 0.991 |
| C22:5N6 | Docosapentaenoate | 29.67 | 79 | y=0.0434x + 0.00343 | 0.993 |
| C22:6N3 | Docosahexaenoate | 30.85 | 79 | y=0.05x + 0.00549 | 0.996 |
| C23:0 | Tricosanoate | 25.92 | 79 | y=0.0327x - 5e-05 | 0.992 |
| C24:0 | Lignocerate | 28.45 | 79 | y=0.051x + 0.00034 | 0.993 |
| C24:1 | Nervonoate | 29.12 | 74 | y=0.0368x + 0.0058 | 0.997 |

Table S3 Concentration of fatty acids (mean±SD, n=3)

| Abbreviation | Name | Concentration (μg/mL) |
| --- | --- | --- |
| C6:0 | Caproate | 26.98±0.39 |
| C8:0 | Caprylate | 30.8±0.67 |
| C10:0 | Caprate | 6.28±0.13 |
| C12:0 | Laurate | 12.68±0.11 |
| C14:0 | Myristate | 84.89±1.8 |
| C14:1T | Myristelaidate | 53.56±5.69 |
| C14:1 | Myristoleate | 46.4±8.99 |
| C15:0 | Pentadecanoate | 19.76±0.56 |
| C15:1T | 10-Transpentadecenoate | 15.19±2.4 |
| C15:1 | 10-Pentadecenoate | 35.64±4.67 |
| C16:0 | Palmitate | 7358.05±289.55 |
| C16:1T | Palmitelaidate | 38.46±12.4 |
| C16:1 | Palmitoleate | 14.81±8.62 |
| C17:0 | Heptadecanoate | 87.79±13.12 |
| C17:1T | 10-Transsheptadecenoate | 52.88±14.29 |
| C17:1 | 10-Heptadecenoate | 24.48±11.6 |
| C18:0 | Stearate | 5155.03±826.27 |
| C18:1N9T | Elaidate | 17547.85±3191.5 |
| C18:1N7T | Transvaccenate | 89.15±26.51 |
| C18:1N9C | Oleate | 13891.98±2846.72 |
| C18:1N7 | Vaccenate | 8.12±3.05 |
| C18:2N6T | Linoelaidate | 9122.81±83.59 |
| C19:1N12T | 7-Transnonadecenoate | 18.06±1.93 |
| C18:2N6 | Linoleate | 1090.58±166.49 |
| C20:0 | Arachidate | 17.88±6.56 |
| C18:3N6 | Gamma Linolenate | 50.74±5.27 |
| C20:1T | Trans 11-Eicosenoate | 1648.59±166.87 |
| C20:1 | 11-Eicosenoate | 324.38±26.06 |
| C18:3N3 | Alpha Linolenate | 65.12±22.41 |
| C21:0 | Heneicosanoate | 88.45±23.69 |
| C20:2 | 11-14 Eicosadienoate | 11.66±6.31 |
| C22:0 | Behenate | 25.22±9.19 |
| C20:3N6 | Homogamma Linolenate | 18.3±9.74 |
| C22:1N9T | Brassidate | 14.18±6.89 |
| C22:1N9 | Erucate | 26.04±5.59 |
| C20:3N3 | 11-14-17 Eicosatrienoate | 323.9±45.41 |
| C20:4N6 | Arachidonate | 49.43±20.78 |
| C23:0 | Tricosanoate | 53.87±25.21 |
| C22:2 | Docosadienoate | 26.91±12.95 |
| C20:5N3 | Eicosapentaenoate | 21.82±8.68 |
| C24:0 | Lignocerate | 21.16±11.01 |
| C24:1 | Nervonoate | 32.96±12.81 |
| C22:4 | Docosatetraenoate | 33.56±14.88 |
| C22:5N6 | Docosapentaenoate | 33.18±5.21 |
| C22:5N3 | Docosapentaenoate | 88.64±10.43 |
| C22:6N3 | Docosahexaenoate | 147.58±44.77 |

Table S4 Antibodies details

| Antibodies | Dilution | Cat No. | Supplier |
| --- | --- | --- | --- |
| LC3A/B Rabbit Antibody | 1：3000 | 4108 | Cell Signaling Technology |
| ULK1 (D8H5) Rabbit mAb | 1：3000 | 8054 | Cell Signaling Technology |
| Beclin-1 (D40C5) Rabbit mAb | 1：3000 | 3495 | Cell Signaling Technology |
| GAPDH (D16H11) XP® Rabbit mAb | 1：3000 | 5174 | Cell Signaling Technology |
| Goat Anti-rabbit IgG (HRP) | 1：3000 | ab97051 | Abcam |
| Goat Anti-Mouse IgG (HRP) | 1：3000 | ab6789 | Abcam |
| mTOR (7C10) Rabbit mAb | 1：3000 | 2983 | Cell Signaling Technology |
| Phospho-mTOR (Ser2448) (D9C2) XP® Rabbit mAb | 1：3000 | 5536 | Cell Signaling Technology |
| Akt (pan) (C67E7) Rabbit mAb | 1：3000 | 4691 | Cell Signaling Technology |
| Phospho-Akt (Ser473) (D9E) XP® Rabbit mAb | 1：3000 | 4060 | Cell Signaling Technology |
| PI3 Kinase P85 Alpha Monoclonal Antibody | 1：3000 | 60225-1-Ig | proteintech |
| Rabbit polyclonal to PI 3 Kinase p85 alpha (phospho Y607) | 1：1000 | ab182651 | Abcam |
| S6 Ribosomal Protein (5G10) Rabbit mAb | 1：1000 | 2217 | Cell Signaling Technology |
| Phospho-S6 Ribosomal Protein (Ser235/236) (2F9) Rabbit mAb | 1：1000 | 4856 | Cell Signaling Technology |
| 4E-BP1 (53H11) Rabbit mAb | 1：1000 | 9644 | Cell Signaling Technology |
| Phospho-4E-BP1 (Thr37/46) (236B4) Rabbit mAb | 1：1000 | 2855 | Cell Signaling Technology |

Table S5 Primers for real-time PCR

| Gene name |  | Primer sequence | Product length (bp) |
| --- | --- | --- | --- |
|  |  |  |  |
| *LC3* | Sense | AACATGAGCGAGTTGGTCAAG | 127 |
|  | Antisense | GCTCGTAGATGTCCGCGAT |  |
| *Beclin-1* | Sense | GCTGCCGTTATACTGTTCT | 180 |
|  | Antisense | CCTCCTGTGTCTTCAATCTT |  |
| *ATG5* | Sense | TGGGCCATCAATCGGAAACTCA | 129 |
|  | Antisense | TGCAGCCACAGGACGAAACAG |  |
| *ATG13* | Sense | TCCAGGCTCGGCTTGGTGAA | 130 |
|  | Antisense | TCCAGGCTCGGCTTGGTGAA |  |
| *p62* | Sense | CTGGCGGAGCAGATGAGGAAGA | 246 |
|  | Antisense | TGGCGGGAGATGTGGGTACAAG |  |
| *ULK1* | Sense | TCTGCCTGTCGTCCACTGTGAA | 148 |
|  | Antisense | GTGATGCTGTGAATGCGGTCCA |  |
| *GAPDH* | Sense | TATGACAACAGCCTCAAGAT | 104 |
|  | Antisense | AGTCCTTCCACGATACCA |  |
